# Quantitative Profiling of Human Milk Oligosaccharides Across Asian Countries Reveals Secretor-Dependent Variations and Implications for Infant Nutrition

**DOI:** 10.3390/ijms27083690

**Published:** 2026-04-21

**Authors:** My Tuyen T. Nguyen, Eun-Hye Kang, Nari Seo, Chang Uk Lim, Ayeon Woo, Yebin An, Seung Yeon Baek, Khanh Hong T. Hoang, Ji A. Jung, Dan Li, Xuan Hong M. To, Beenish Israr, Hyun Joo An, Jaehan Kim

**Affiliations:** 1 College of Pharmacy, University of Michigan, Ann Arbor, MI 48108, USA; mytuyen1108@gmail.com; 2 Biopharmaceutical & Biotherapeutic Practice Training Center, Chungnam National University, Daejeon 34134, Republic of Korea; ehkang@cnu.ac.kr; 3 Graduate School of Analytical Science and Technology, Chungnam National University, Daejeon 34134, Republic of Korea; seonari85@gmail.com (N.S.); hjan@cnu.ac.kr (H.J.A.); 4 Department of Food and Nutrition, Chungnam National University, Daejoen 34134, Republic of Korea; imchanguk05@gmail.com (C.U.L.); woozn01@gmail.com (A.W.); ahnyebin99@gmail.com (Y.A.); yeoni@cnu.ac.kr (S.Y.B.); 5 Major of Glocal Life-Care Convergence, Chungnam National University, Daejeon 34134, Republic of Korea; 6 Interdisciplinary Education Center for the Innovative Next Generation Leaders in Glocal Lifecare, Chungnam National University, Daejeon 34134, Republic of Korea; 7 Faculty of Food, Nutrition and Home Science, University of Agriculture, Faisalabad 38000, Pakistan; beenish_israr@hotmail.com; 8 Department of Nutrition and Food Science, Industrial University of Ho Chi Minh City, 12 Nguyen Van Bao, Hanh Thong Ward, Ho Chi Minh City 700000, Vietnam; hong_01259005@iuh.edu.vn; 9 Maeil Asia Human Milk Research Center, Maeil Dairies Co., Ltd., 63 Jinwiseo-ro, Jinwi-myeon, Pyeongtaek 17706, Republic of Korea; asonsum@gmail.com; 10 College of Food Science and Engineering, Changchun University, Changchun 130022, China; drlidan@yahoo.com; 11 Department of Obstetrics and Gynecology, University of Medicine and Pharmacy at Ho Chi Minh City, Ho Chi Minh City 700000, Vietnam; tomaixuanhong@ump.edu.vn

**Keywords:** human milk oligosaccharides, Asian populations, secretor status, fucosylation, mass spectrometry, infant nutrition, geographical variation

## Abstract

Human milk oligosaccharides (HMOs) exhibit substantial inter-individual and secretor-dependent variation, yet comprehensive quantitative data across diverse maternal phenotypes remain limited. In this study, we analyzed 578 human milk samples from four Asian populations using a dual mass spectrometry approach, combining quadrupole time-of-flight (Q-TOF) for structural profiling and triple quadrupole (QQQ) mass spectrometry for absolute quantitation of 15 major HMOs. Samples were classified into Secretor (76.7%) and Non-Secretor (23.3%) groups based on α-1,2-fucosylated HMO profiles. Secretor milk was enriched in α-1,2-fucosylated HMOs, whereas Non-Secretor milk showed markedly reduced levels of these structures. However, Non-Secretor retained substantial total fucosylated HMOs (65–76% of Secretor levels), accompanied by increased α-1,3/4-fucosylated structures, including up to 3.2-fold higher levels of 3-fucosyllactose (3-FL). Sensitive QQQ quantitation further revealed trace levels of α-1,2-fucosylated HMOs in Non-Secretor at concentrations 10–100-fold lower than in Secretor. Correlation analysis indicated an inverse relationship between α-1,2- and α-1,3-fucosylation patterns, consistent with redistribution of fucosylation pathways. These findings suggest that the Non-Secretor phenotype represents a distinct compositional state rather than a simple loss of α-1,2-fucosylation and provide a quantitative framework for phenotype-informed nutritional strategies.

## 1. Introduction

Human milk oligosaccharides (HMOs) are a structurally diverse group of unconjugated glycans and represent the third-most abundant solid component in human milk after lactose and lipids. Their concentrations typically range from 5 to 20 g/L, with over 200 distinct structures identified to date [1,2,3]. Owing to their unique glycosidic linkages, HMOs resist digestion by gastric and intestinal enzymes and reach the colon largely intact [4]. Although not directly utilized as a nutrient source by the infant, HMOs play critical roles in shaping gut microbiota composition, modulating immune development, and protecting against pathogens [5,6].

The structural diversity of HMOs originates from their composition of five monosaccharide building blocks: D-glucose, D-galactose, N-acetylglucosamine, L-fucose, and N-acetylneuraminic acid [5]. These oligosaccharides are built upon a lactose core that can be elongated through β-1,3 linkages to form lacto-N-biose (type I chains) or β-1,6 linkages to form N-acetyllactosamine (type II chains). These core structures are further diversified by the addition of fucose residues through α-1,2-, α-1,3-, and α-1,4-linkages, and/or sialic acid residues via α-2,3- and α-2,6-linkages [3,7,8,9]. The biosynthesis of these modifications is mediated by specific glycosyltransferases, whose expression and activity vary among individuals and across populations.

The concentrations and profiles of HMOs are dynamic and are influenced by multiple factors, including lactational age and maternal genetics (e.g., *FUT2* and *FUT3* polymorphisms). It is well established that the total abundance of HMOs declines as lactation progresses from the transitional to the mature stage [10,11]. While lactational age largely determines overall concentration levels, genetic factors play a major role in shaping HMO composition. In particular, secretor status is associated with differences in fucosylation patterns, including the relative abundance of α-1,2- and α-1,3/4-fucosylated structures, which tend to be maintained across lactational stages [11,12].

Among genetic factors influencing HMO composition, maternal secretor status is recognized as a major determinant. The secretor (Se) gene, *FUT2*, encodes α-1,2-fucosyltransferase 2, which governs the synthesis of α-1,2-fucosylated HMOs [13]. Non-Secretor mothers, who carry loss-of-function mutations in *FUT2*, produce milk with markedly reduced levels of α-1,2-fucosylated structures such as 2′-fucosyllactose (2′-FL) and lacto-N-fucopentaose I (LNFP I) [12]. Global population studies report Secretor frequencies ranging from 72% to 89%, with corresponding Non-Secretor frequencies of 9% to 22% [14,15,16,17,18,19,20]. Given that human milk represents the primary source of nutrition during early infancy, these genetically determined differences in HMO composition may influence microbiota development and host–microbe interactions. However, the functional implications of these differences remain incompletely understood, particularly with respect to whether distinct HMO profiles in Non-Secretor milk represent a deficiency or an alternative compositional state shaped by compensatory biosynthetic processes.

Beyond genetic factors, variation in HMO profiles has also been observed across populations and geographical regions, reflecting differences in glycosyltransferase gene polymorphisms, dietary patterns, and environmental influences [21]. However, the majority of existing HMO data has been derived from Western populations, with comprehensive analyses of Asian cohorts remaining limited. Available studies suggest potential differences in HMO composition between Asian and Western populations, yet systematic comparisons across multiple Asian countries are still lacking. This gap is particularly important given that Asia represents over 60% of the global population and encompasses substantial genetic, dietary, and environmental diversity.

Comprehensive characterization of HMOs presents significant analytical challenges due to their structural complexity, wide concentration range (from mg/L to g/L), and extensive isomeric diversity. Early studies primarily relied on high-performance liquid chromatography with fluorescence detection or single mass spectrometry platforms, which limited either structural coverage or quantitative accuracy [22,23]. Recent advances in mass spectrometry have enabled more integrated approaches combining qualitative profiling with absolute quantitation. Quadrupole time-of-flight (Q-TOF) mass spectrometry provides high-resolution structural information through accurate mass measurement and fragmentation analysis [24], whereas triple quadrupole (QQQ) mass spectrometry offers superior sensitivity and precision for targeted quantitation [25]. A dual mass spectrometry strategy leveraging the complementary strengths of these platforms offers a powerful approach for comprehensive and quantitative analysis of HMOs at the population level.

The recent commercial availability of major HMOs, including 2′-FL, 3-FL, 3′-SL, 6′-SL, 3′-GL, and 6′-GL, has created new opportunities for evidence-based supplementation in infant formula [26,27]. However, the effective application of these advances requires a clear understanding of natural variation in HMO composition across maternal phenotypes and populations. Establishing quantitative reference ranges is essential for interpreting the biological significance of these compositional differences and for guiding future nutritional strategies. At present, the lack of comprehensive quantitative HMO datasets for Asian populations limits the ability to define population-specific baselines and constrains the broader applicability of HMO research.

To address these knowledge gaps, the present study employed a dual mass spectrometry approach combining Q-TOF profiling with QQQ-based absolute quantitation to comprehensively characterize HMO composition in a large cohort of Asian mothers. Human milk samples (*n* = 578) were collected from four Asian countries—Korea, Yanbian (China), Vietnam, and Pakistan—representing diverse genetic backgrounds and dietary patterns. The specific objectives were to: (1) characterize HMO structural profiles using Q-TOF mass spectrometry and compare patterns across populations; (2) perform absolute quantitation of major HMOs using QQQ mass spectrometry with authenticated standards to establish concentration ranges; (3) compare HMO composition between Secretor and Non-Secretor, including detection of low-abundance α-1,2-fucosylated structures; and (4) examine correlations among HMO structures to explore biosynthetic relationships. Accordingly, this study focuses primarily on genotype-associated variation in HMO composition, particularly differences related to maternal secretor status, rather than direct geographical comparisons. Population diversity is used as a framework to evaluate whether these patterns are consistently observed across distinct Asian cohorts.

This work provides a comprehensive and systematically characterized dataset of HMO composition in Asian populations and demonstrates an integrated analytical approach for large-scale glycomic studies.

## 2. Results

### 2.1. Comprehensive Profiling of HMO Composition Across Asian Countries by Liquid Chromatography–Quadrupole Time-of-Flight Mass Spectrometry (LC–Q-TOF-MS)

**Figure 1:**
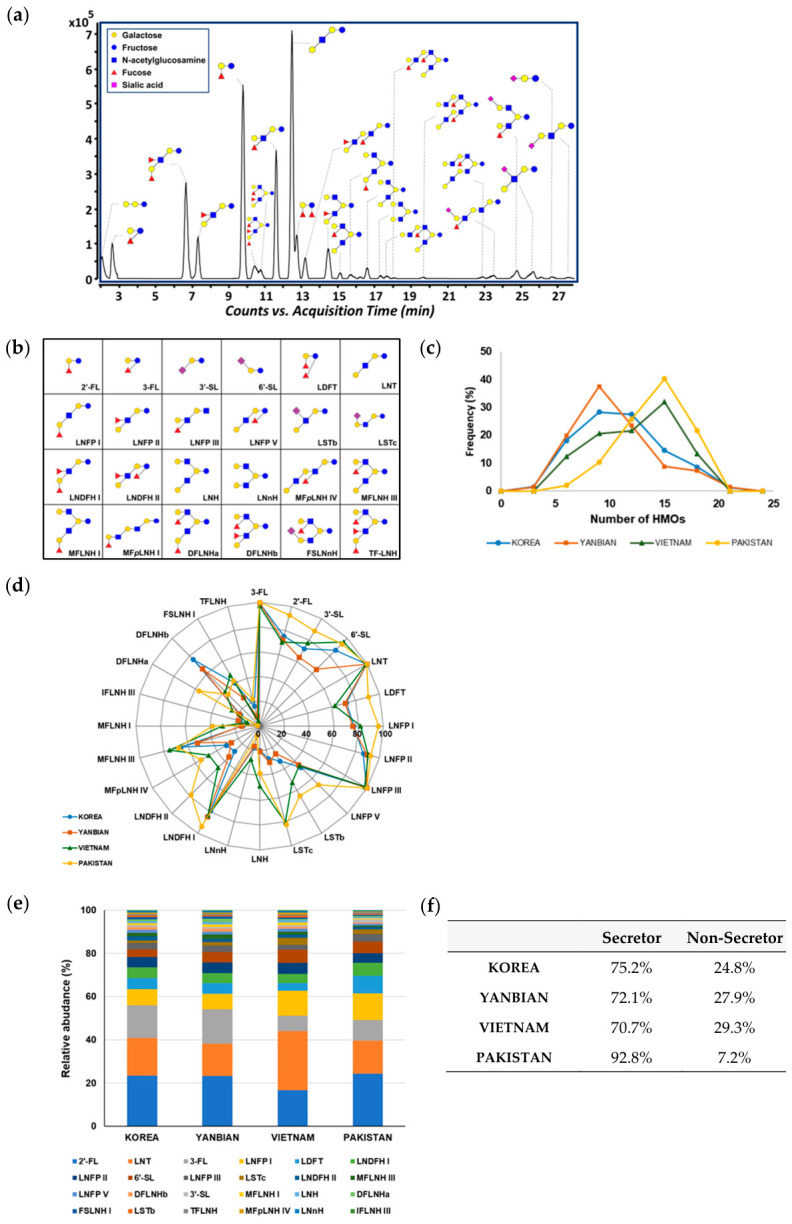

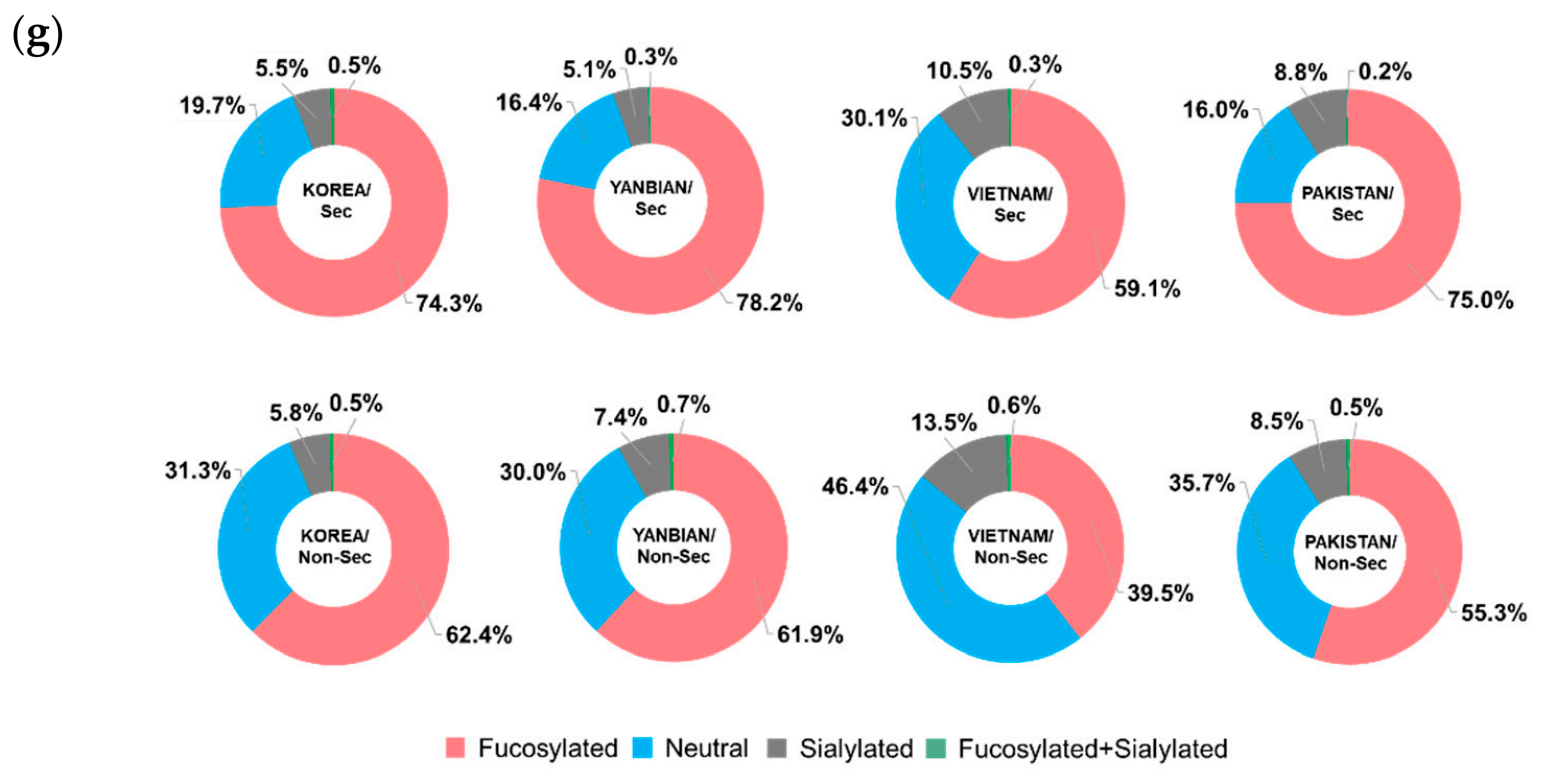
Profile of oligosaccharides in Asian human milk by LC-QTOF mass spectrometry; (**a**) Chromatogram of Korean mothers’ milk oligosaccharides; (**b**) Structures of major HMOs in Asian countries; (**c**) Number of identified HMO structures by Asian countries; (**d**) Frequency of individual HMO structures across countries; (**e**) Relative intensity of each oligosaccharide; (**f**) Distribution of Secretor and Non-Secretor across populations; (**g**) Composition of HMOs by structural class across countries and secretor status.

Over 100 HMO compounds were detected in Asian human milk by Q-TOF mass spectrometry. On average, approximately 50–60 distinct compositions were identified in each country. The degree of polymerization (DP) of HMO structures ranged from 3 to 9. A representative chromatogram illustrating the major HMOs in pooled Korean milk is presented in Figure 1a. Through comprehensive structural analysis combining accurate mass measurement and MS/MS fragmentation patterns, 24 significant HMOs commonly found across all four Asian populations were identified and structurally characterized (Figure 1b).

Figure 1c illustrates the frequency distribution of the total number of HMO structures detected in human milk from the Asian countries. Among individuals from each population, those from Yanbian (China) represent a geographically distinct Korean diaspora population with a shared genetic background but different dietary patterns. Korean and Yanbian samples typically contained 9–15 dominant HMOs, while Pakistani samples exhibited a broader distribution with 15 to 21 dominant HMOs. Vietnamese samples showed high variability, with HMO structure numbers ranging from 9 to 21, reflecting considerable inter-individual diversity within this population.

Among the 24 identified HMOs, several core structures such as 3-FL, LNT, and LNFP III were consistently detected across most samples regardless of country (Figure 1d). In addition, a subset of HMOs, including 2′-FL, LNFP I–III, LDFT, LNDFH I, 3′-SL, and 6′-SL, was frequently observed across all populations, suggesting their roles as common components of Asian milk oligosaccharide profiles. Notably, LSTc exhibited marked population-dependent variation in detection frequency. It was more frequently detected in Vietnamese and Pakistani samples, whereas its occurrence was substantially lower in Korean and Yanbian cohorts, indicating population-specific differences in sialylation patterns.

Analysis of relative abundance revealed that 12 major HMOs (2′-FL, LNT, 3-FL, LNFP I, LDFT, LNDFH I, LNFP II, 3′-SL, 6′-SL, LNFP III, LSTc, and LSTb) collectively accounted for the majority of total HMO signal intensity, consistent with previous observations that a limited number of structures dominate overall HMO composition (Figure 1e). The relative abundance patterns observed in Korean, Yanbian, and Pakistani samples were broadly similar, with 2′-FL, LNT, and 3-FL consistently representing the most abundant HMOs. In contrast, Vietnamese samples exhibited a distinct profile characterized by a relatively higher contribution of LNT compared to other populations, consistent with Non-Secretor-associated compositional patterns.

The frequency of α-1,2-fucosylated HMOs (2′-FL, LNFP I, LDFT, and LNDFH I) served as reliable indicators of maternal secretor status within each population. Samples lacking detectable signals for 2′-FL, LDFT, and LNFP I under Q-TOF analytical conditions were classified as originating from Non-Secretor.

Figure 1f presents the distribution of Secretor and Non-Secretor phenotypes across the Asian populations studied. Overall, 23.3% of the cohort was classified as Non-Secretor, with relatively consistent ratios observed in Korea (24.8%), Yanbian (27.9%), and Vietnam (29.3%). In contrast, Pakistan demonstrated a markedly lower Non-Secretor frequency of only 7.2%, suggesting population-specific differences in *FUT2* genotype distribution.

The composition of HMOs by structural class exhibited pronounced differences between Secretor and Non-Secretor (Figure 1g). Fucosylated HMOs dominated in Secretor, comprising approximately 60–80% of total HMOs, compared to 40–60% in Non-Secretor.

Among the populations, Vietnamese samples exhibited the most pronounced proportional differences between phenotypes. In contrast, sialylated HMOs maintained relatively consistent proportions across both Secretor and Non-Secretor, indicating that sialylation is independent of the *FUT2* genotype. Fucosylated–sialylated HMOs represented a minor fraction (<1%) and showed no phenotype dependence.

These Q-TOF profiling data established the foundation for subsequent targeted quantitative analysis of major HMO structures.

### 2.2. Absolute Quantitation of Major HMOs and Secretor-Dependent Variations by Liquid Chromatography–Triple Quadrupole Mass Spectrometry (LC-QQQ-MS/MS)

**Figure 2:**
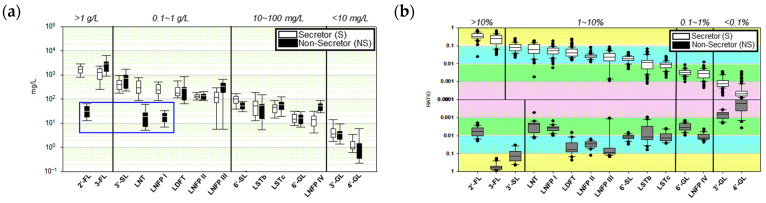

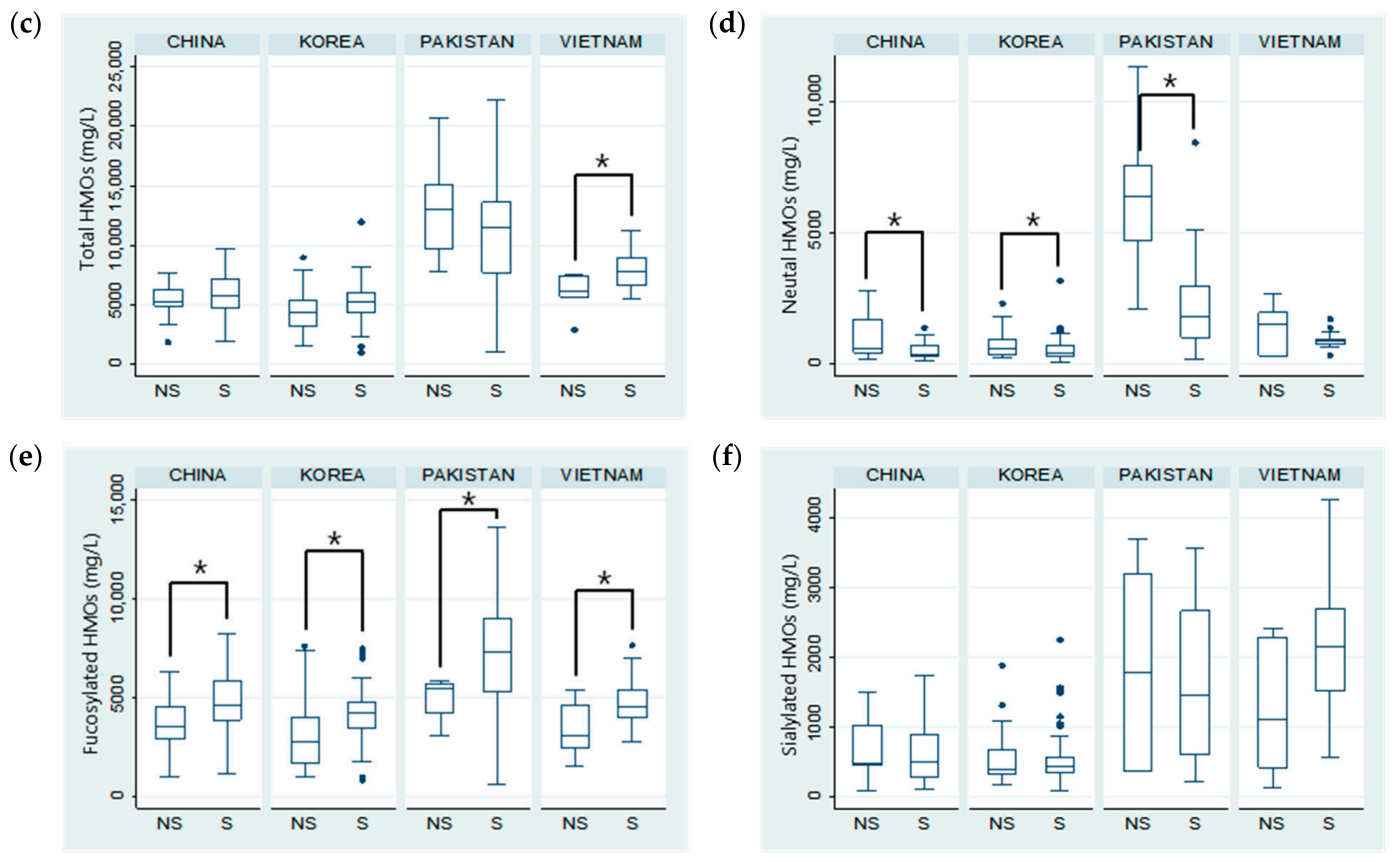
Quantitative profiling of HMOs by country and secretor status. (**a**) Representative dynamic range of HMO concentrations (mg/L). The blue boxes in (**a**) highlight the HMO components that are significantly lower in the non-secretor group compared to the secretor group.; (**b**) Relative abundance of individual HMOs expressed as a percentage of total HMOs; (**c**) Total HMO content across four countries; (**d**) Neutral HMOs; (**e**) Fucosylated HMOs; (**f**) Sialylated HMOs across four countries. Abbreviations: S, Secretor; NS, Non-Secretor. Significant differences between non-secretor (NS) and secretor (S) groups are indicated by * *p* < 0.05.

Based on Q-TOF profiling, 15 major HMO structures representing more than 80% of total signal intensity were selected for absolute quantitation by LC-QQQ-MS/MS using authenticated reference standards: 2′-FL, 3-FL, LNT, LNFP I, LDFT, 6′-SL, 3′-SL LNFP II, LNFP III, LSTb, LSTc, 6′-GL, LNFP V, 3′-GL, and 4′-GL. It should be noted that differences between Q-TOF-derived relative intensities (Figure 1) and LC–QQQ–MS/MS-based absolute quantitation (Figure 2) are attributable to platform-specific analytical characteristics. Q-TOF provides semi-quantitative profiling, whereas LC–QQQ–MS/MS enables accurate quantitation using external standards. Thus, variations in relative proportions between the two datasets reflect methodological differences rather than biological inconsistencies.

Figure 2a presents a representative profile of Korean mothers’ milk, illustrating the substantial dynamic range spanning four orders of magnitude. Secretor’s HMO concentrations fell into four distinct tiers: (1) 2′-FL and 3-FL exceeded 1 g/L (>10% of total); (2) LNT, LNFP I, LDFT, 6′-SL, 3′-SL, and LNFP II ranged from 0.1 to 1 g/L (1–10% of total); (3) LNFP III, LSTb, LSTc, 6′-GL, and LNFP V fell between 10 and 100 mg/L; and (4) 3′-GL and 4′-GL were below 10 mg/L (Figure 2b).

Ternary plot analysis revealed distinct compositional differences between Secretor and Non-Secretor phenotypes, with each shown in the left and right panels, respectively (Supplementary Figure S1). Secretor clustered within a relatively confined compositional space characterized by high fucosylation and low sialylation, indicating a consistent compositional profile. In contrast, Non-Secretor exhibited broad dispersion across the ternary space, reflecting greater variability in HMO composition. This wide scattered distribution suggests substantially increased inter-individual heterogeneity among Non-Secretor, likely associated with alternative fucosylation pathways. Neutral HMO proportion showed generally similar distributions between phenotypes in most populations, although wider variability was observed in Pakistani and Vietnamese Non-Secretor.

#### 2.2.1. Total HMO Concentration

Before interpreting absolute HMO concentrations, it is essential to consider differences in lactational age among the studied cohorts (Supplementary Table S1). Samples from the Korean (111.8 ± 68.6 days) and Yanbian cohorts (132.5 ± 104.0 days) primarily represented mature milk, whereas the samples from Vietnam (43.2 ± 64.1 days) and Pakistan (46.1 ± 46.1 days) were collected at earlier lactation stages. As lactational age is a major determinant of baseline HMO concentrations, the population-level summaries presented below inherently reflect these temporal variations. Therefore, absolute inter-cohort comparisons should be interpreted with caution and considered primarily as contextual rather than direct biological differences.

Total HMO concentrations varied substantially across countries, ranging from 5.0 to 11.6 g/L (Table 1 and Figure 2c). The Korean and Yanbian cohorts showed comparable levels (5.0–5.8 g/L), while the Vietnamese cohort exhibited moderately higher concentrations (~7.4 g/L). The Pakistani cohort displayed exceptionally high HMO levels (11.6 ± 5.1 g/L), approximately two-fold higher than those observed in the Korean and Yanbian cohorts. In light of these baseline differences, mechanistic interpretations in this study are derived primarily from phenotype-stratified analyses (Secretor vs. Non-Secretor), focusing on consistent HMO redistribution patterns associated with secretor status.

Secretor status had minimal impact on total HMO concentrations in most populations. The Korean and Yanbian cohorts showed comparable levels between Secretor and Non-Secretor (4.5–6.0 g/L range, Table 1). Similarly, the Pakistani cohort maintained high concentrations in both phenotypes despite the elevated baseline (11.1–13.2 g/L range, Figure 2c). Although total HMOs in Vietnamese Non-Secretor were lower (6.0 g/L) compared to Secretor (7.9 g/L), the overall magnitude of difference remained moderate relative to the baseline concentration. These population-level differences are presented descriptively, whereas mechanistic interpretations throughout this study are derived primarily from Secretor phenotype-stratified analyses. Consequently, conclusions regarding biosynthetic regulation are not dependent on absolute inter-country concentration differences.

#### 2.2.2. Fucosylated HMOs

**Figure 3:**
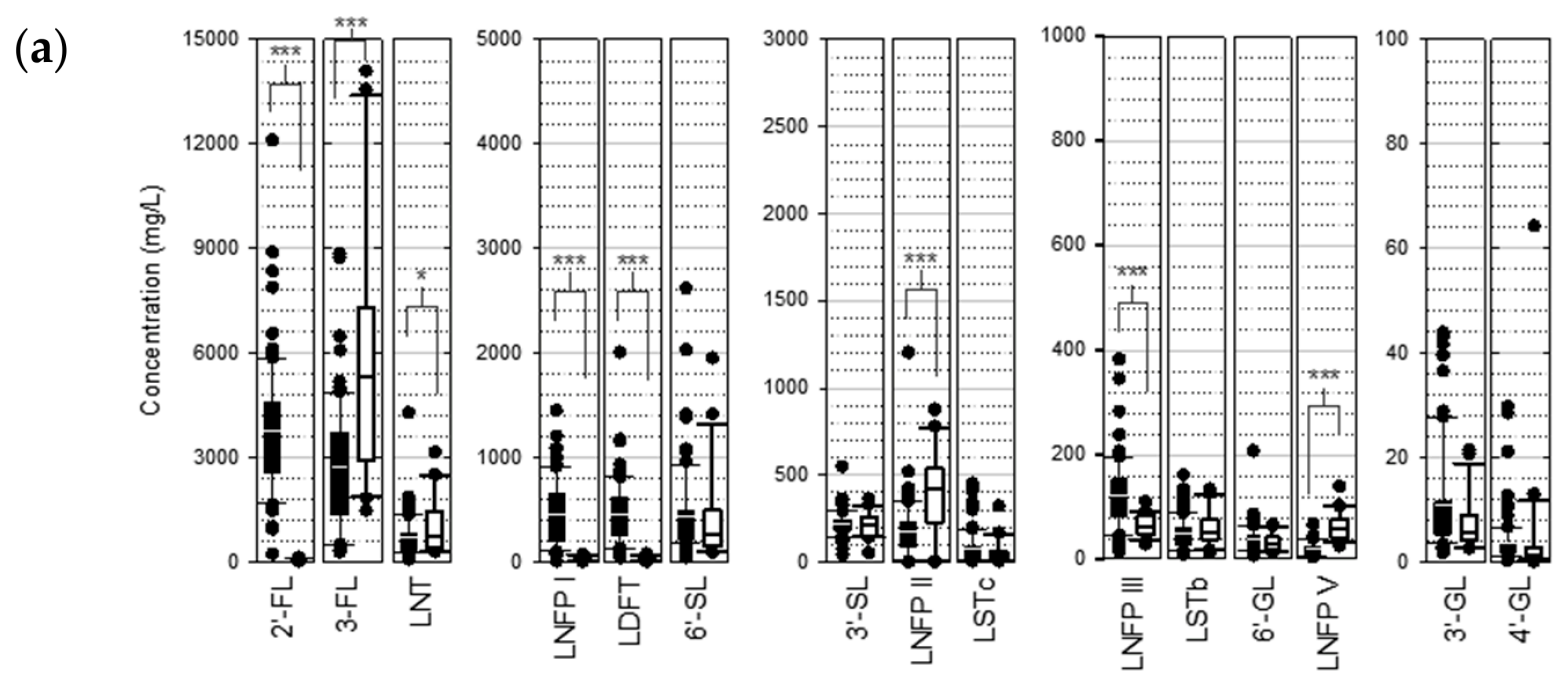

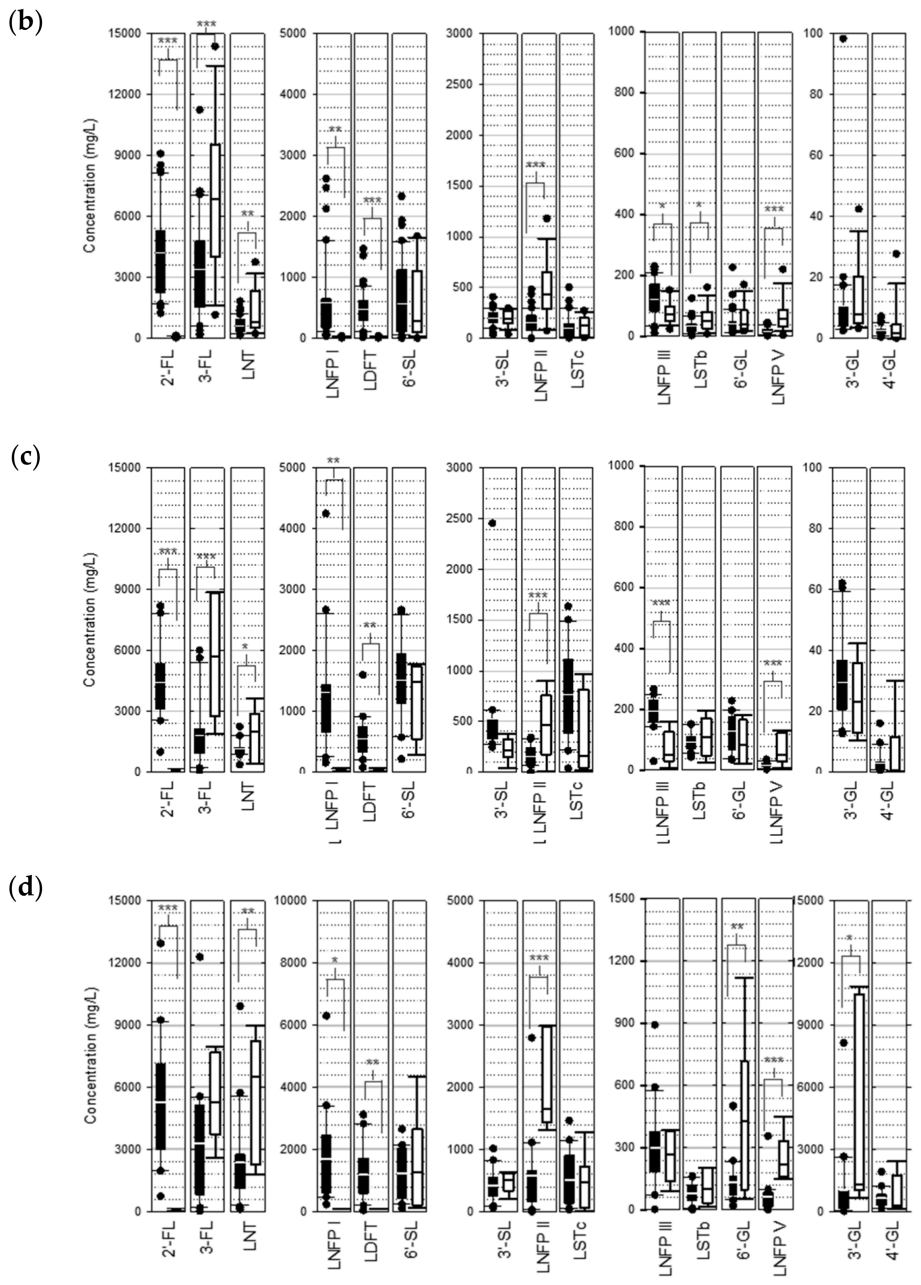
Box and whisker plots of 15 major HMOs from Secretor (Black box) and Non-secretor (white box) in Asian human milk (mg/L). (**a**) Korea; (**b**) Yanbian; (**c**) Vietnam; (**d**) Pakistan. Significant differences between Secretor and Non-secretor groups are indicated by * *p* < 0.05, ** *p* < 0.01, and *** *p* < 0.001.

Fucosylated HMOs (2′-FL, 3-FL, LDFT, LNFP I, LNFP II, LNFP III, and LNFP V) ranged from 3.9 to 6.7 g/L across populations (Table 1 and Figure 3). Korean samples exhibited the lowest levels (3.9 g/L, 77.8% of total HMOs), while Yanbian and Vietnamese samples showed comparable absolute concentrations (4.5–4.6 g/L). Pakistani samples displayed the highest absolute concentration (6.7 g/L) but the lowest relative proportion (57.6%) due to elevated neutral HMOs.

Secretor and Non-Secretor differed significantly in fucosylated HMO concentrations (Figure 2e and Figure 3), although Non-Secretor retained appreciable levels. Median concentrations in Non-Secretor ranged from 2.7 to 5.5 g/L—corresponding to approximately 65–75% of Secretor levels (4.2–7.3 g/L). As proportions of total HMOs, fucosylated structures comprised 37.5–71.0% in Non-Secretor versus 61.3–81.3% in Secretor (Table 1), indicating that overall fucosylated HMO abundance is partially maintained despite the loss of α-1,2-fucosylation.

Secretor contained high concentrations of α-1,2-fucosylated markers: 2′-FL (1.9–2.6 g/L), LDFT (0.3–0.8 g/L), and LNFP I (0.4–1.5 g/L) (Table 1 and Figure 3). Notably, these “secretor-specific” structures were not completely absent in Non-Secretor. Although undetectable by Q-TOF analysis, sensitive QQQ quantitation revealed their presence at trace levels in Non-Secretor (Figure 3 and Figure S3), with concentrations approximately 10–100-fold lower than in Secretor.

Conversely, Non-Secretor exhibited elevated α-1,3/4-fucosylated structures (Table 1 and Figure 3). For example, 3-FL concentrations in Non-Secretor (2.7–3.0 g/L) exceeded those in Secretor (0.9–1.7 g/L), and similar trends were observed for LNFP II and LNFP V. These patterns suggest a consistent redistribution of fucosylation toward α-1,3/4-linked structures in the absence of FUT2 activity, consistent with altered distribution of fucosylation patterns.

#### 2.2.3. Neutral HMOs

Neutral HMOs (LNT, 3′-GL, 4′-GL, and 6′-GL) showed striking population-dependent variation (Table 1, Figure 2d and Figure 3). Korean and Yanbian samples contained similar levels (0.6 g/L, 10.5–11.4% of total), while Vietnamese samples exhibited intermediate concentrations (1.0 g/L, 13.5% of total). Pakistani samples exhibited substantially elevated neutral HMO levels (3.2 g/L, 27.9% of total). This increase was primarily driven by higher concentrations of LNT (2.2 g/L) and 3′-GL (0.9 g/L), compared to 0.5–0.9 g/L and 0.005–0.014 g/L, respectively, in other populations (Table 1 and Figure 3). 

Non-Secretor consistently exhibited higher neutral HMO levels across all populations, exceeding those observed in Secretor. The most pronounced difference was observed in Pakistan, where Non-Secretor reached 6.4 g/L compared to 2.3 g/L in Secretor (Figure 3). Although the Vietnamese cohort showed high inter-individual variability, Non-Secretor still tended to exhibit elevated neutral HMO levels. Notably, LNT emerged as the predominant HMO in Pakistani Non-Secretor, and significant differences between Secretor and Non-Secretor were consistently observed across populations (Figure 3).

#### 2.2.4. Sialylated HMOs

Sialylated HMOs (3′-SL, 6′-SL, LSTb, and LSTc) exhibited marked population-dependent variation (Table 1, Figure 2f and Figure 3). Vietnamese samples showed the highest sialylated content (1.9 g/L, 26.1% of total), whereas Korean and Yanbian samples showed the lowest levels (0.5–0.6 g/L, 10.8–11.1% of total). Pakistani samples showed intermediate levels (1.7 g/L, 14.5% of total). The elevated sialylation observed in Vietnamese and Pakistani samples was primarily driven by higher LSTc abundance, which was approximately 5–9-fold higher than in Korean and Yanbian samples (Figure 3).

Notably, secretor status had minimal impact on sialylated HMO concentrations (Figure 2f and Figure 3), with similar levels observed between Secretor and Non-Secretor within each population. The only exception was LSTb in the Yanbian cohort, which showed differences between phenotypes; however, high residual standard deviation (RSD = 75.4%) in Non-Secretor suggested that this difference is likely driven by inter-individual variation rather than a consistent phenotype effect. These findings demonstrate that sialylation pathways are largely independent of the *FUT2* genotype and are primarily influenced by population-specific factors.

### 2.3. Quantitative Correlation Analysis Reveals Two Distinct Biosynthetic Groups

**Figure 4:**
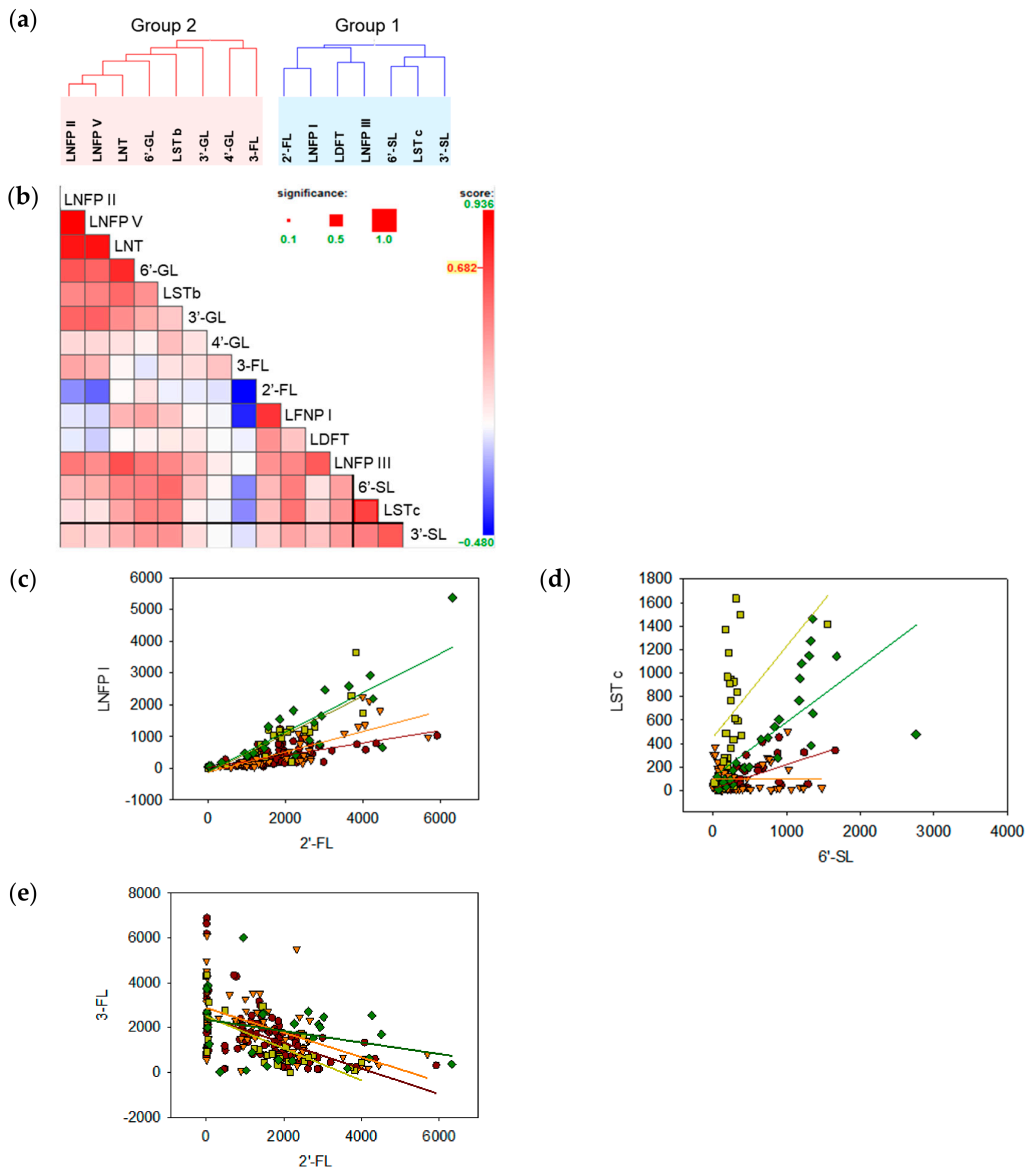
Pearson correlation between HMOs using absolute quantitation data. (**a**) Dendrogram; (**b**) Pearson correlation matrix; (**c**–**e**) scatter plots show the linear regression between HMOs from four Asian countries. Data points are color-coded by country: Korea (Red circles), Yanbian (Orange inverted triangles), Vietnam (Yellow squares), and Pakistan (Green diamonds). The solid lines represent the linear regression fits (mean trends) for each respective group.

Hierarchical clustering analysis based on absolute quantitation data revealed two major groups of structurally related HMOs (Figure 4a). Group (i) comprised α-1,2-fucosylated HMOs (2′-FL, LDFT, and LNFP I) along with sialylated structures (3′-SL, 6′-SL, and LSTc), which predominated in Secretor. Group (ii) consisted of α-1,3-fucosylated HMOs (3-FL, LNFP II, and LNFP V) and neutral HMOs (LNT, 3′-GL, 6′-GL, and 4′-GL), which were more abundant in Non-Secretor. Within group (i), the sialylated HMOs formed a distinct subcluster, indicating strong inter-correlation among these structures independent of Secretor.

The correlation matrix confirmed positive correlations within each group, with Pearson correlation coefficients (r) ranging from 0.5 to 0.9 (Figure 4b and Table 2). Strong correlations were observed between 2′-FL and other α-1,2-fucosylated structures (LDFT; r ≈ 0.3–0.5, LNFP I; r = 0.73), as well as among sialylated HMOs (e.g., 6′-SL and LSTc; r ≈ 0.7). These patterns were consistent across all populations (Supplementary Tables S3–S6), suggesting coordinated biosynthetic behavior across populations. Representative scatter plots illustrate key relationships between major HMOs (Figure 4c–e and Figure S4). The positive correlation between 2′-FL and LNFP I (r = 0.7–0.8) is consistent with their shared dependence on FUT2 activity. Similarly, the correlation between 6′-SL and LSTc (r = 0.6–0.8) suggests coordinated sialylation of lactose- and LNT-derived structures.

Notably, 3-FL exhibited a negative correlation with 2′-FL across the dataset (r = −0.48, Table 2 and Figure 4e), which was consistently observed across individual populations (r = −0.30 to −0.70; Supplementary Tables S3–S6). This inverse relationship is consistent with potential competition between *FUT2*- and *FUT3*-mediated pathways utilizing shared substrates. In Secretor, FUT2 activity is associated with higher levels of α-1,2-fucosylation, whereas in Non-Secretor, reduced FUT2 activity is accompanied by increased α-1,3-fucosylation. Together, these findings support a model of redistribution in fucosylation pathways rather than direct evidence of enzymatic competition.

### 2.4. Integration of Quantitative and Correlation Data to Illustrate HMO Redistribution Patterns

By integrating the comprehensive Q-TOF profiling, absolute quantitation data, and Pearson correlation analysis, we characterized how maternal secretor status is associated with the overall HMO profile. This integrative approach enabled the identification of specific HMO structures reduced in the Non-Secretor group and provided quantitative support for redistribution patterns that contribute to maintaining HMO diversity across maternal phenotypes. These findings are summarized in a proposed biosynthetic framework (Supplementary Figure S5).

The quantitative results suggest that the synthesis of neutral non-fucosylated and sialylated HMOs proceeds independently of the *FUT2* genotype. Specifically, the positive correlation between 6′-SL and LSTc (r ≈ 0.6–0.7 in several populations) is consistent with coordinated sialylation of lactose- and LNT-derived cores. This pattern suggests that α-2,6-sialyltransferase activity may be influenced more strongly by population-level factors than by secretor-dependent fucosylation status.

In contrast, the biosynthesis of fucosylated HMOs exhibited pronounced Secretor-dependent regulation. Through the integration of Q-TOF frequency data and sensitive QQQ quantitation, structures containing α-1,2-fucosyl linkages—including 2′-FL, LDFT, and LNFP I—were markedly reduced in Non-Secretor. Notably, the negative correlation observed between 2′-FL and 3-FL across countries (r = −0.7 to −0.3) supports differential utilization of shared substrates between FUT2- and FUT3-mediated pathways for shared substrates.

In Non-Secretor lacking functional FUT2, increased relative abundance of α-1,3/4-fucosylated structures (e.g., 3-FL, LNFP II, and LNFP V) was observed. These patterns are consistent with a redistribution of fucosylation pathways in response to reduced α-1,2-fucosylation. Importantly, this redistribution pattern was consistently observed across populations despite variation in lactational stage and population background, suggesting a consistent phenotypic trend associated with non-secretor status.

## 3. Discussion

The benefits of HMOs for breastfed infants—including shaping the intestinal microbiota, modulation of immune responses, and protection against infections—are known to depend on their structural diversity [6,28]. However, HMO composition and concentration vary among mothers due to genetic and lactational factors [10,29]. Therefore, comprehensive characterization of HMO structure and abundance is essential for understanding their biological roles and for informing the development of infant nutrition strategies.

The absolute concentration differences observed across the four populations should be interpreted in the context of variation in lactational stage among cohorts (mature milk in Korea and Yanbian versus earlier stages in Vietnam and Pakistan). However, characterizing these temporal or geographical differences was not the primary objective of this study. Instead, our analysis focused on genotype-associated differences in HMO composition. Notably, the shift toward α-1,3/4-fucosylated structures in Non-Secretor was consistently observed across all populations, suggesting that this phenotype-associated pattern is largely independent of lactational stage.

These findings are consistent with previous large-scale studies in Chinese populations [30], which demonstrated that secretor status is a primary determinant of HMO composition, particularly influencing the balance between α-1,2-fucosylated HMOs (e.g., 2′-FL, LNFP I) and neutral structures such as LNT. In addition, these studies reported substantial lactation-dependent variation in HMO concentrations, supporting our interpretation that baseline differences across populations should be considered in the context of lactational stage rather than as direct biological contrasts.

In this study, we employed a dual mass spectrometry approach combining Q-TOF for comprehensive profiling with LC–QQQ–MS/MS for targeted quantitation. Q-TOF analysis provided broad structural coverage, identifying over 100 HMO species with degrees of polymerization up to 9, while enabling differentiation of isomeric structures based on retention time and fragmentation patterns. This approach enabled phenotypic classification of secretor status without genetic testing and revealed population-dependent structural features that may not be captured by targeted approaches alone [31]. Subsequent QQQ-based quantitation of 15 major HMOs provided absolute concentration data spanning four orders of magnitude (0.01–10 g/L), establishing quantitative reference ranges across populations and phenotypes.

Importantly, the higher sensitivity of QQQ analysis revealed trace levels of α-1,2-fucosylated structures (2′-FL, LDFT, and LNFP I) in Non-Secretor at concentrations 10–100-fold lower than in Secretor samples. These structures were not detectable by Q-TOF but were measurable by QQQ, indicating that the apparent absence of secretor-associated HMOs reflects a quantitative reduction rather than complete absence. This observation has implications for understanding residual enzymatic activity, potential alternative biosynthetic routes, and the functional relevance of low-abundance HMOs.

Across 578 samples from four Asian populations, we observed both conserved and population-dependent features in HMO composition. A core set of structures—including 2′-FL, 3-FL, LNT, LNFP I–III, LDFT, LNDFH I, LSTc, and 6′-SL—accounted for the majority of total HMO abundance (>80%). Beyond this core repertoire, variability in structural diversity and relative abundance patterns was observed, with non-secretor-enriched samples showing a greater contribution from LNT and secretor-enriched samples characterized by 2′-FL dominance.

Absolute quantitation revealed substantial variation in total HMO concentrations across populations, with median values ranging from 5.0 to 5.8 g/L in Korea and Yanbian to higher levels in Vietnam (7.4 g/L) and Pakistan (11.6 g/L). However, phenotype-stratified analyses indicated that secretor status primarily influences the distribution of individual HMO classes rather than total concentration. These findings suggest that inter-population differences in HMO profiles likely reflect a combination of genetic background and environmental influences, although their relative contributions remain to be clarified.

The biological implications of *FUT2* polymorphisms extend beyond milk composition, as Non-Secretor status has been associated with altered susceptibility to certain pathogens such as norovirus and rotavirus [32,33]. At the same time, Secretor milk contains higher levels of α-1,2-fucosylated HMOs that can act as decoy receptors for pathogens [6,26], reflecting a potential biological trade-off between host defense and milk composition.

Despite reduced α-1,2-fucosylated HMOs, Non-Secretor retained substantial levels of total fucosylated HMOs. This was associated with elevated α-1,3/4-fucosylated structures, including 3-FL, LNFP II, and LNFP V, which were consistently higher in Non-Secretor. These patterns reflect a redistribution of fucosylation across Secretor phenotypes toward α-1,3/4-linked structures in response to reduced FUT2 activity. A conceptual framework summarizing these relationships is provided in Supplementary Figure S5.

Correlation analysis further supported this redistribution pattern. The negative correlation between 2′-FL and 3-FL (r = −0.48, *p* < 0.001), consistently observed across populations (r = −0.30 to −0.70), is consistent with a shift in relative pathway utilization. This inverse relationship was also observed within Secretor, where higher levels of 2′-FL were associated with lower levels of 3-FL. Together, these findings support a model in which fucosylation patterns reflect differential allocation of shared substrates across pathways, although direct evidence of enzymatic competition cannot be established from the present data.

Our analysis further suggests that neutral non-fucosylated and sialylated HMOs are largely independent of *FUT2* status, consistent with their similar concentrations across phenotypes. The strong correlation between 6′-SL and LSTc (r = 0.6–0.8) is consistent with coordinated sialylation of lactose- and LNT-derived cores, suggesting that sialylation is less influenced by *FUT2*-dependent fucosylation.

Hierarchical clustering identified two major compositional groups: (i) α-1,2-fucosylated and sialylated HMOs (Secretor-enriched), and (ii) α-1,3/4-fucosylated and neutral HMOs (Non-Secretor-enriched). This separation is consistent with the influence of the FUT2 genotype on HMO composition together with redistribution across fucosylation pathways.

Although α-1,2-fucosylated HMOs such as 2′-FL are markedly reduced in Non-Secretor, α-1,3/4-fucosylated HMOs are relatively elevated, resulting in substantial total fucosylated HMO levels across phenotypes. This phenotypic divergence may reflect underlying biological trade-offs related to host–microbe interactions and environmental factors, rather than a simple deficiency in HMO composition.

Together, our results demonstrate that FUT2 status is associated with systematic changes in HMO composition through altered redistribution across fucosylation pathways, rather than a simple loss of fucosylation, preserving overall structural diversity.

Accordingly, caution is warranted when translating these findings into supplementation strategies. While the commercial availability of HMOs offers opportunities for personalized nutrition, supplementing Non-Secretor infants with 2′-FL to mimic the Secretor profile assumes that the Secretor phenotype is universally superior, which has not yet been definitively established. Future studies should focus on linking distinct HMO profiles with clinical outcomes, such as microbiome development and infection susceptibility, to enable evidence-based and phenotype-informed nutritional strategies.

## 4. Materials and Methods

### 4.1. Standards and Chemicals

The following standards were purchased from Carbosynth Ltd. (Berkshire, UK): 2′-fucosyllactose (2′-FL), 3-fucosyllactose (3-FL), 4′-galactosyllactose (4′-GL), 6′-galactosyllactose (6′-GL), 3′-sialyllactose (3′-SL), 6′-sialyllactose (6′-SL), and sialyl-lacto-N-tetraose c (LSTc). Lactodifucotetraose (LDFT), lacto-N-tetraose (LNT), and lacto-N-fucopentaose I (LNFP I) were obtained from ProZyme, Inc. (Hayward, CA, USA).

HPLC-grade acetonitrile was procured from Sigma-Aldrich (St. Louis, MO, USA), while formic acid and trifluoroacetic acid were obtained from Fisher Scientific (Pittsburgh, PA, USA). Porous graphitic carbon cartridges (Bond Elut Carbon, 250 mg) were sourced from Agilent Technologies (Santa Clara, CA, USA).

### 4.2. Sample Collection

Human milk samples were collected from South Korea, China (Yanbian Korean Autonomous Prefecture), Vietnam (Ho Chi Minh City), and Pakistan (Faisalabad). The Yanbian cohort was included as a comparative population, as it shares genetic similarities with the Korean population while differing in environmental and dietary factors.

A total of 578 samples were collected from healthy mothers (Korea, *n* = 252; Yanbian, *n* = 137; Vietnam, *n* = 92; Pakistan, *n* = 97). Samples were collected without pre-selection based on maternal diet, supplementation, or nutritional status, to capture representative variability within each population.

The samples represent different stages of lactation, with mean postpartum times of 111.8 ± 68.6 days (Korea) and 132.5 ± 104.0 days (Yanbian, China), corresponding primarily to mature milk. In contrast, samples from Vietnam (43.2 ± 64.1 days) and Pakistan (46.1 ± 46.1 days) were collected at earlier lactation stages. Detailed subject characteristics are provided in Supplementary Table S1.

All procedures were approved by the Institutional Review Boards of Chungnam National University (Korea); (201612-BR-062-01; 5 December 2016), Maeil Dairies Co., Ltd. (Korea); (0627-201306-HRBR-001-02; 27 June 2013), the University of Medicine and Pharmacy at Ho Chi Minh City (Vietnam); (IRB-VN01002; 19 December 2016), and the University of Agriculture at Faisalabad (Pakistan); (IORG0005731; 27 March 2018). Written informed consent was obtained from all participants.

The sample collection method was described in detail in our previous study [34]. Briefly, human milk was directly expressed into sterilized 50 mL conical tubes. Samples were immediately transported to the laboratory in well-insulated containers with gel ice packs to maintain low temperature. All human milk samples were stored at −80 °C prior to analysis.

Milk samples were collected at a single postpartum time point for each subject. Although lactational stage influences absolute HMO concentrations, the present study focused on relative compositional patterns and genotype-associated redistribution. Therefore, analyses emphasized phenotype-stratified comparisons rather than direct comparison of absolute concentrations across populations.

### 4.3. Sample Preparation for LC-MS/MS

#### 4.3.1. HMO Extraction

The extraction and purification of HMOs were conducted following previously described methods [22,23]. Recovery was evaluated using the standard addition method for each batch. Neutral HMOs showed recovery rates of 92–100%, while sialylated HMOs showed recovery rates of 82–85%. Concentrations of sialylated HMOs were collected using compound-specific recovery factors. Matrix effects were not significant under the experimental conditions.

#### 4.3.2. Standard Preparation

HMO standards were reduced and purified by solid-phase extraction (SPE) as described above. A mixed standard solution containing 2′-FL, 3-FL, 4′-GL, 6′-GL, 3′-SL, 6′-SL, LSTc, LDFT, LNT, and LNFP I was prepared at 100 mg/L. Following cleanup, calibration curves were prepared over the range of 1–2000 μg/L for most HMOs. For 2′-FL and 3-FL, calibration ranges of 0.1–10 mg/L were used due to their higher abundance in human milk.

All calibration curves showed excellent linearity (r^2^ = 0.994–0.999). The limit of quantitation (LOQ) was defined as a signal-to-noise ratio ≥ 10 and ranged from 1 to 5 μg/L. Coefficients of variation (CV) were below 15% for all analytes.

#### 4.3.3. Profiling of HMOs in Human Milk by Liquid Chromatography–Mass Spectrometry

Purified samples were analyzed using an Agilent 6540 Accurate-Mass LC-QTOF-MS system (Agilent Technologies, Santa Clara, CA, USA). Chromatographic separation was performed on a porous graphitized carbon column (Hypercarb™, 3 μm, 2.1 × 100 mm; Thermo Scientific, Pittsburgh, PA, USA). The column temperature was maintained at 40 °C. The mobile phase consisted of (A) water with 0.1% formic acid and (B) acetonitrile with 0.1% formic acid. The flow rate was 0.3 mL/min with a 55 min gradient. Mass spectrometry was performed in positive ion mode over an m/z range of 300–2000. The average mass accuracy was within 10 ppm.

#### 4.3.4. Quantitative Analysis of Target HMOs by LC-ESI-MS^2^

Quantitative analysis was performed using an Agilent 6470 triple quadrupole LC-MS/MS system (Agilent Technologies, Santa Clara, CA, USA). Chromatographic conditions were similar to those used for Q-TOF analysis.

HMOs were analyzed in positive ion mode using multiple reaction monitoring (MRM). MRM transitions and parameters are provided in Supplementary Table S2 and Figure S2.

### 4.4. Data Analysis

#### 4.4.1. HMO Identification

Q-TOF data were processed using Agilent MassHunter Qualitative Analysis software (version B.07.01 SP1; Agilent Technologies, Santa Clara, CA, USA). Peaks were extracted using the “Find by Molecular Features” algorithm and matched against an in-house library based on accurate mass and retention time. Detected ions included [M + H]^+^ and [M + 2H]^2+^ species. Structural assignments were confirmed using MS/MS fragmentation patterns.

#### 4.4.2. Data Processing

Relative abundance was calculated as the percentage of each HMO signal relative to the total signal intensity. Absolute concentrations were obtained from QQQ calibration curves. LNFP II, LNFP III, and LNFP V were quantified using LNFP I calibration, while LSTb was quantified using LSTc. Prior to correlation analysis, data were log-transformed where appropriate to reduce skewness. Pearson correlation coefficients were calculated using Hierarchical Clustering Explorer 3.5 (Human-Computer Interaction Lab, University of Maryland, College Park, MD, USA). Group comparisons were performed using one-way ANOVA followed by Scheffé’s post hoc test (*p* < 0.05). Statistical analyses were conducted using Stata/SE version 12.1 (StataCorp LLC, College Station, TX, USA) and SigmaPlot 12.5 (Systat Software Inc., Palo Alto, CA, USA).

## Figures and Tables

**Table 1:** Absolute concentration of HMOs in Asian human milk (mg/L).

		Average			Median			Percentile		
		Total	Sec	Non-Sec	Total	Sec	Non-Sec	Total	Sec	Non-Sec
Total	KOREA	5042.2 ± 1616.3	5199.0 ± 1533.0	4503.8 ± 1808.4	1.0	1.0	0.7	[0.8, 1.6]	[0.9, 1.7]	[0.3, 1.5]
	YANBIAN	5821.4 ± 1803.6	5997.1 ± 1850.6	5267.3 ± 1585.5	1.0	1.1	0.7	[0.6, 1.5]	[0.7, 1.5]	[0, 1.9]
	VIETNAM	7420.7 ± 1807.9	7860.1 ± 1628.7 ^b^	5956.0 ± 1707.7 ^a^	0.7	0.7	0.2	[0.4, 1.7]	[0.4, 1.6]	[0.1, 2.0]
	PAKISTAN	11,568.6 ± 5079.3	11,072.8 ± 5234.4	13,221.2 ± 4540.8	1.6	1.9	1.0	[1.0, 2.8]	[1.1, 2.7]	[0.7, 4.1]
Fucosylated	KOREA	3921.8 ± 1415.5	4132.8 ± 1174.2 ^b^	3197.2 ± 1897.7 ^a^	15.2	14.8	15.8	[10.6, 23.3]	[11.2, 23.7]	[9.7, 22.5]
	YANBIAN	4565.2 ± 1588.9	4878.4 ± 1478.9 ^b^	3577.4 ± 1571.2 ^a^	19.0	19.2	18.7	[11.4, 28.7]	[8.7, 24.2]	[13.1, 42.3]
	VIETNAM	4477.5 ± 1418.6	4817.9 ± 1263.5 ^b^	3343.1 ± 1409.8 ^a^	65.5	68.1	42.7	[29.9, 87.7]	[33.9, 88.4]	[14.4, 77.7]
	PAKISTAN	6667.5 ± 2999.2	7181.6 ± 3210.7 ^b^	4953.8 ± 1105.7 ^a^	58.3	53.8	215.0	[42.5, 100.3]	[39.6, 78.2]	[87.2, 282.2]
Neutral	KOREA	577.0 ± 462.2	525.9 ± 419.1 ^a^	752.5 ± 562.6 ^b^	3.4	3.6	2.8	[2.5, 5.4]	[2.7, 5.7]	[2.4, 4.1]
	YANBIAN	609.4 ± 517.3	484.3 ± 303.7 ^a^	1004.1 ± 807.6 ^b^	4.0	3.9	4.3	[2.1, 6.6]	[2.1, 5.3]	[2.8, 9.4]
	VIETNAM	1003.7 ± 537.3	894.0 ± 292.9	1369.4 ± 953.3	12.0	12.5	11.6	[10.4, 16.7]	[10.6, 16.8]	[8.0, 15.8]
	PAKISTAN	3225.1 ± 2840.7	2271.0 ± 1994.7 ^a^	6405.4 ± 3073.9 ^b^	332.9	295.9	648.7	[127.7, 642.1]	[96.9, 499]	[602.3, 4065.2]
Sialyl- ated	KOREA	543.4 ± 366.6	540.3 ± 356.2	554.0 ± 408.7	437.7	401.9	540.8	[262.1, 692.9]	[251.4, 599.7]	[299.8, 879.7]
	YANBIAN	646.8 ± 428.8	634.4 ± 423.7	685.7 ± 459.9	436.5	328.1	555.0	[243.8, 728.9]	[233.2, 657.9]	[398.9, 1635.2]
	VIETNAM	1939.4 ± 1001.0	2148.2 ± 930.9	1243.6 ± 980.6	776.5	752.1	1424.8	[654.1, 1132.3]	[674.8, 837.9]	[508.8, 1800.8]
	PAKISTAN	1676.0 ± 1134.1	1620.2 ± 1080.7	1862.0 ± 1392.4	1639.7	992.6	4587.0	[883.4, 2917.2]	[811.4, 1794.4]	[2220.4, 5579.3]
2′-FL	KOREA	1442.5 ± 1100.1	1853.1 ± 899.9 ^b^	32.2 ± 18.5 ^a^	1541.4	1786.3	25.9	[490.8, 1981.3]	[1331.9, 2200.7]	[18.3, 44.5]
	YANBIAN	1610.6 ± 1368.4	2105.3 ± 1198.9 ^b^	50.5 ± 82.0 ^a^	1302.8	2038.3	28.5	[638.9, 2439.5]	[1203.5, 2502.3]	[18.8, 34.9]
	VIETNAM	1670.0 ± 1207.2	2162.2 ± 901.3 ^b^	29.3 ± 24.0 ^a^	1775.2	1973.9	18.6	[672.4, 2412.9]	[1555.1, 2549.6]	[17.4, 40.9]
	PAKISTAN	1992.8 ± 1678.2	2576.9 ± 1466.0 ^b^	45.8 ± 21.7 ^a^	1714.5	2448.3	44.0	[510.9, 2923.9]	[1481.5, 3177.8]	[32.4, 49.8]
3-FL	KOREA	1628.1 ± 1266.3	1323.7 ± 861.0 ^a^	2673.6 ± 1804.9 ^b^	1363.4	1273.5	2158.6	[785.3, 2007.4]	[716.8, 1807.2]	[1128.5, 3410.5]
	YANBIAN	1993.0 ± 1385.1	1685.5 ± 1153.7 ^a^	2962.9 ± 1641.3 ^b^	1669.9	1473.3	2429.5	[1014.4, 2628.4]	[947.2, 2336.8]	[2102, 4211.2]
	VIETNAM	1311.7 ± 1237.1	872.8 ± 775.1 ^a^	2775.0 ± 1424.6 ^b^	797.1	702.3	2800.8	[485.0, 1606]	[453.7, 1015.4]	[1725.2, 4021.1]
	PAKISTAN	1847.4 ± 1403.5	1601.8 ± 1431.9 ^a^	2666.1 ± 1006.1 ^b^	2011.5	1603.3	2565.6	[583.8, 2585.8]	[472.6, 2486.8]	[2113.6, 3463.4]
LDFT	KOREA	236.2 ± 206.3	299.0 ± 193.2 ^b^	20.3 ± 10.2 ^a^	202.9	250.0	17.9	[55.4, 349.2]	[175.9, 394.2]	[13.4, 29.2]
	YANBIAN	239.5 ± 205.9	307.8 ± 190.4 ^b^	24.2 ± 14.0 ^a^	232.3	278.4	17.8	[47.9, 331.7]	[195.1, 397.9]	[15.4, 26.6]
	VIETNAM	269.3 ± 234.9	347.1 ± 212.2 ^b^	10.2 ± 6.1 ^a^	258.5	296.5	10.2	[66.4, 344.2]	[221.0, 419.5]	[5.6, 13.1]
	PAKISTAN	586.9 ± 588.8	756.1 ± 570.6 ^b^	23.0 ± 10.5 ^a^	441.9	635.8	20.7	[60.3, 728.9]	[397.7, 900.6]	[14.3, 30.7]
LNFP I	KOREA	306.3 ± 284.1	390.2 ± 269.8 ^b^	18.2 ± 18.0 ^a^	212.2	289.9	9.6	[68, 496]	[178.5, 555.3]	[7.2, 20]
	YANBIAN	389.7 ± 536.8	508.3 ± 567.3 ^b^	15.8 ± 14.4 ^a^	178.6	265.0	13.0	[51.1, 394.6]	[153, 557.9]	[7.8, 17.5]
	VIETNAM	870.9 ± 833.9	1127.5 ± 784.1 ^b^	15.8 ± 16.6 ^a^	985.7	1143.4	12.4	[147.2, 1208.9]	[681.3, 1225.5]	[6.0, 14.1]
	PAKISTAN	1133.1 ± 1225.1	1451.0 ± 1230.3 ^b^	73.5 ± 11.9 ^a^	705.5	1101.8	77.1	[244.1, 1628.7]	[599.4, 1917.7]	[63.1, 82.4]
LNFP II	KOREA	189.5 ± 174.9	144.7 ± 138.7 ^a^	343.4 ± 200.8 ^b^	148.9	116.9	364.1	[81.2, 251.6]	[74.7, 188.3]	[234.8, 421.1]
	YANBIAN	208.0 ± 203.7	147.7 ± 138.8 ^a^	398.1 ± 259.9 ^b^	147.8	117.3	360.4	[68.5, 264.2]	[64.8, 202.3]	[266.7, 545.3]
	VIETNAM	190.3 ± 185.0	128.5 ± 83.0 ^a^	396.4 ± 280.6 ^b^	117.4	98.9	398.3	[69.1, 241]	[68.5, 172.8]	[222.9, 575.7]
	PAKISTAN	770.3 ± 758.0	486.9 ± 527.0 ^a^	1715.1 ± 652.1 ^b^	497.2	388.1	1412.3	[219.2, 1072.6]	[168.4, 541.0]	[1282.9, 2276.6]
LNFP III	KOREA	95.2 ± 53.7	106.8 ± 55.0 ^b^	55.2 ± 18.0 ^a^	84.5	101.1	53.0	[57.3, 124.2]	[76, 130.5]	[41.4, 69]
	YANBIAN	97.3 ± 49.5	107.4 ± 49.9 ^b^	65.4 ± 33.0 ^a^	87.1	107.9	58.5	[61.6, 134.3]	[75.6, 146.4]	[43.5, 83.2]
	VIETNAM	140.9 ± 63.9	165.1 ± 46.0 ^b^	60.3 ± 47.6 ^a^	149.7	174.9	44.7	[123.2, 192.5]	[135.2, 196.1]	[35.0, 84.6]
	PAKISTAN	246.1 ± 153.9	254.5 ± 167.5	218.4 ± 103.4	238.6	238.6	226.1	[156.3, 320.6]	[167.7, 317.9]	[145.6, 307.7]
LNFP V	KOREA	24.1 ± 21.3	15.3 ± 9.1 ^a^	54.4 ± 23.4 ^b^	16.9	14.1	56.3	[9.2, 32.2]	[7.9, 19.9]	[35.1, 66.7]
	YANBIAN	27.1 ± 31.6	16.4 ± 12.6 ^a^	60.6 ± 47.8 ^b^	15.2	13.3	49.4	[8.6, 32.6]	[8.3, 20.9]	[29.9, 73.1]
	VIETNAM	24.3 ± 26.9	14.8 ± 8.0 ^a^	56.0 ± 42.5 ^b^	17.0	12.6	42.3	[10.1, 27.0]	[9.9, 19.2]	[32.3, 89.5]
	PAKISTAN	90.7 ± 98.0	54.4 ± 64.9 ^a^	211.9 ± 95.5 ^b^	58.1	44.1	186.8	[27.6, 114.5]	[19.8, 72.2]	[145.2, 242]
LNT	KOREA	551.8 ± 452.2	500.0 ± 407.3 ^a^	729.7 ± 554.7 ^b^	132.3	132.0	148.8	[114.6, 154.6]	[115.1, 152.3]	[108.6, 174.4]
	YANBIAN	578.2 ± 500.2	455.7 ± 290.0 ^a^	964.4 ± 782.4 ^b^	138.7	135.0	143.0	[99.8, 168]	[100.1, 165.3]	[92.6, 171.2]
	VIETNAM	926.5 ± 522.8	812.4 ± 282.1 ^a^	1307.0 ± 915.7 ^b^	244.9	280.4	135.9	[182.3, 299.0]	[209.3, 316.7]	[120.9, 183.9]
	PAKISTAN	2213.2 ± 1964.0	1670.1 ± 1589.0 ^a^	4023.8 ± 2139.5 ^b^	251.8	227.1	317.3	[191.0, 366.9]	[177.5, 330.1]	[244.7, 383.9]
3′-GL	KOREA	5.0 ± 4.7	5.4 ± 5.1	3.7 ± 2.5	175.6	178.3	170.5	[125.7, 307.6]	[135.9, 307.5]	[102.1, 314.5]
	YANBIAN	5.7 ± 7.2	5.3 ± 7.6	6.9 ± 6.0	192.4	188.4	218.3	[70.5, 667.3]	[72.4, 636]	[69.7, 677.7]
	VIETNAM	14.3 ± 7.0	14.9 ± 7.3	12.3 ± 6.0	927.4	968.5	723.4	[557, 1151.5]	[718.9, 1233.3]	[261.2, 1046.2]
	PAKISTAN	905.3 ± 1530.3	531.8 ± 912.4 ^a^	2150.3 ± 2475.4 ^b^	793.4	767.7	793.4	[271.3, 1277.6]	[271.9, 1226.9]	[291.1, 1205.6]
4′-GL	KOREA	2.0 ± 3.2	1.8 ± 2.6	2.5 ± 6.7	46.4	45.2	47.6	[31.3, 59.9]	[29.8, 59.4]	[35.8, 70.2]
	YANBIAN	1.4 ± 2.0	1.2 ± 0.8	2.0 ± 3.8	29.6	28.2	50.2	[17.9, 53.6]	[17.7, 43.5]	[28.4, 77.2]
	VIETNAM	1.8 ± 3.2	1.5 ± 1.9	3.1 ± 6.0	92.5	92.5	109.1	[64.3, 117.4]	[71.9, 105.6]	[55.8, 162.7]
	PAKISTAN	2.3 ± 1.9	2.2 ± 1.5	2.7 ± 3.2	77.6	74.3	101.6	[42.6, 119.7]	[43.5, 113.9]	[54.5, 175.1]
6′-GL	KOREA	18.2 ± 12.1	18.6 ± 13.1	16.7 ± 8.0	48.4	54.1	36.5	[21.0, 76.7]	[23.9, 83.8]	[15.4, 58.1]
	YANBIAN	24.2 ± 21.0	22.0 ± 20.0	30.9 ± 23.5	31.7	28.7	39.4	[13.8, 165.8]	[13.6, 135]	[14.2, 190]
	VIETNAM	61.1 ± 31.6	65.3 ± 29.8	47.1 ± 36.4	601.4	724.4	157.2	[276.2, 938.2]	[415.0, 999.2]	[55.4, 634.8]
	PAKISTAN	104.2 ± 120.3	66.9 ± 51.6 ^a^	228.5 ± 195.4 ^b^	408.7	329.6	463.3	[129.8, 739.9]	[157.8, 813.9]	[163.3, 527.1]
3′-SL	KOREA	137.9 ± 47.0	135.4 ± 44.2	146.4 ± 55.8	454.9	438.1	551.1	[279.1, 708.5]	[274.9, 644.5]	[316.5, 923.0]
	YANBIAN	140.0 ± 69.0	140.2 ± 71.6	139.6 ± 62.6	464.5	356.3	570.9	[255.6, 765.2]	[241.1, 683.2]	[424.2, 1671.3]
	VIETNAM	285.7 ± 272.6	328.9 ± 296.3	141.8 ± 75.2	859.3	846.0	1494.3	[734.4, 1188.1]	[750.6, 933.6]	[530.1, 1910.0]
	PAKISTAN	274.4 ± 146.2	266.6 ± 157.8	300.3 ± 105.9	2074.0	1820.0	6401.4	[1028.3, 4879.2]	[992.8, 2898.2]	[5078.8, 7310.5]
6′-SL	KOREA	282.3 ± 452.3	277.7 ± 258.4	298.2 ± 320.0	3884.7	4225.6	2768.8	[3162.8, 4674.7]	[3473.7, 4750.9]	[1751, 3906.7]
	YANBIAN	370.8 ± 391.6	366.7 ± 398.3	383.7 ± 385.1	4419.7	4639.6	3532.6	[3673.3, 5483.1]	[3864.4, 5856.5]	[2886.6, 4541.2]
	VIETNAM	884.1 ± 445.0	957.5 ± 418.0	639.2 ± 482.2	4322.4	4562.3	3078.3	[3720.5, 5211.6]	[3969.0, 5355.3]	[2554.3, 4254.6]
	PAKISTAN	818.8 ± 637.4	770.0 ± 516.8	981.8 ± 987.5	6338.0	7301.1	5456.1	[4995.7, 8580.3]	[5499.4, 8929.3]	[4468.8, 5670.2]
LSTb	KOREA	50.2 ± 30.3	48.6 ± 29.7	56.0 ± 32.3	425.1	427.8	398.9	[340.1, 568.8]	[350.8, 567.7]	[326.6, 597.1]
	YANBIAN	39.7 ± 30.9	34.1 ± 23.5 ^a^	57.4 ± 43.9 ^b^	486.1	486.8	464.2	[316.9, 940]	[283.3, 885.8]	[451.5, 1013.9]
	VIETNAM	96.7 ± 42.2	92.7 ± 30.1	110.1 ± 71.9	2111.7	2152.6	1116.0	[1127, 2487.1]	[1544.5, 2687]	[482.7, 2105.2]
	PAKISTAN	86.0 ± 55.8	78.9 ± 47.7	109.6 ± 78.0	1608.5	1455.7	1778.7	[584.4, 2686.2]	[607.8, 2645.7]	[691.5, 2865.4]
LSTc	KOREA	73.0 ± 85.2	78.7 ± 88.9	53.4 ± 69.4	5068.6	5164.9	4274.9	[4122.4, 5913.7]	[4292.1, 5960.9]	[3438.2, 5312.8]
	YANBIAN	96.3 ± 113.6	93.5 ± 118.1	105.1 ± 102.1	5663.9	5765.3	5215.1	[4671.2, 7073.4]	[4618.5, 7136.5]	[4841.4, 6250.6]
	VIETNAM	673.0 ± 482.0	769.1 ± 468.2	352.5 ± 410.6	7474.9	7817.3	6161.3	[6423.6, 8083.6]	[6643.6, 8792.6]	[5685, 7162.6]
	PAKISTAN	496.8 ± 436.5	504.8 ± 443.1	470.4 ± 453.0	11,906.5	11,484.0	13,053.4	[8652.3, 13,713.4]	[8000.2, 13,524.2]	[10,342.9, 14,737.5]

^a,b^ Values are presented as mean ± SD. Different superscript letters (a, b) within the same row indicate statistically significant differences between Secretor and Non-Secretor groups (*p* < 0.05).

**Table 2:** Pearson correlation coefficients between HMO concentrations in Asian human milk (*n* = 208; subset used for correlation analysis).

	4′-GL	6′-GL	3′-GL	LNT	2′-FL	3-FL	LDFT	LNFP I	LNFP II	LNFP III	LNFP V	3′-SL	6′-SL	LSTb	LSTc
4′-GL	1.00														
6′-GL	0.04	1.00													
3′-GL	0.10	0.30	1.00												
LNT	0.11	0.76	0.42	1.00											
2′-FL	−0.05	0.11	−0.03	0.02	1.00										
3-FL	0.23	−0.04	0.12	0.03	−0.48	1.00									
LDFT	0.00	0.04	0.09	0.01	0.39	−0.03	1.00								
LNFP I	−0.02	0.32	0.03	0.27	0.73	−0.40	0.21	1.00							
LNFP II	0.14	0.62	0.56	0.83	−0.21	0.33	−0.04	−0.04	1.00						
LNFP III	0.07	0.49	0.27	0.63	0.39	0.00	0.60	0.44	0.50	1.00					
LNFP V	0.14	0.57	0.57	0.84	−0.29	0.28	−0.09	−0.07	0.94	0.42	1.00				
3′-SL	0.00	0.38	0.12	0.29	0.17	−0.06	0.22	0.33	0.19	0.36	0.16	1.00			
6′-SL	0.03	0.47	0.22	0.44	0.26	−0.23	0.11	0.47	0.26	0.35	0.30	0.47	1.00		
LSTb	0.23	0.40	0.21	0.55	−0.03	0.10	0.08	0.21	0.44	0.43	0.46	0.39	0.54	1.00	
LSTc	−0.02	0.46	0.05	0.32	0.23	−0.22	0.17	0.52	0.12	0.38	0.10	0.61	0.68	0.46	1.00

## Data Availability

The original contributions presented in this study are included in the article/Supplementary Material. Further inquiries can be directed to the corresponding author(s).

## Supplementary Materials

The following supporting information can be downloaded at https://www.mdpi.com/article/10.3390/ijms27083690/s1.

## Abbreviations

The following abbreviations are used in this manuscript:

2′-FL	2′-Fucosyllactose
3-FL	3-Fucosyllactose
3′-GL	3′-Galactosyllactose
3′-SL	3′-Sialyllactose
4′-GL	4′-Galactosyllactose
6′-GL	6′-Galactosyllactose
6′-SL	6′-Sialyllactose
DFLNHa	Difucosyl-lacto-N-hexaose a
DFLNHb	Difucosyl-lacto-N-hexaose b
FSLNnH	Fucosyl-sialyl-lacto-N-neohexaose
FUT2	Fucosyltransferase 2
FUT3	Fucosyltransferase 3
IFLNH III	Isomeric-fucosylated-lacto-N-Hexaose III
LDFT	Lactodifucotetraose
LNDFH I	Lacto-N-difucohexaose I
LNDFH II	Lacto-N-difucohexaose II
LNFP I	Lacto-N-fucopentaose I
LNFP II	Lacto-N-fucopentaose II
LNFP III	Lacto-N-fucopentaose III
LNFP V	Lacto-N-fucopentaose V
LNH	Lacto-N-hexaose
LNnH	Lacto-N-neohexaose
LNT	Lacto-N-tetraose
LSTb	Sialyl-lacto-N-tetraose b
LSTc	Sialyl-lacto-N-tetraose c
MFLNH I	Monofucosyl-lacto-N-hexaose I
MFLNH III	Monofucosyl-lacto-N-hexaose III
MF*p*LNH IV	Monofucosyl-*para*-lacto-N-hexaose IV
MF*p*LNnH I	Monofucosyl-*para*-lacto-N-neohexaose I
TFLNH	Trifucosyl-lacto-N-hexaose
