# Supplementary material for: Quantitative Profiling of Human Milk Oligosaccharides Across Asian Countries Reveals Secretor-Dependent Variations and Implications for Infant Nutrition
Source: Int J Mol Sci. 2026 Apr 21;27(8):3690. doi: 10.3390/ijms27083690 (PMC13116441; doi:10.3390/ijms27083690)
Supplement: Supplementary file 1 [file ijms-27-03690-s001.zip › ijms-4116244-supplementary.pdf]

Quantitative Profiling of Human Milk Oligosaccharides Across Asian Countries Reveals Secretor-Dependent Variations and Implications for Infant Nutrition

My Tuyen T. Nguyen [1,†], Eun-Hye Kang [2,†], Nari Seo [3], Chang Uk Lim [4,5,6], Ayeon Woo [4,5,6], Yebin An [4,5,6], Seung Yeon Baek [4,11], Khanh Hong T. Hoang [7], Ji A. Jung [8], Dan Li [9], Xuan Hong M. To [10], Beenish Israr [11], Hyun Joo An [3] and Jaehan Kim [4,5,6,11,*,†]

Supplementary Figures and Tables

Supplementary Figure S1.

Quantitative distribution of glycan groups, The left ternary plots represent Secretor, and the right plots represent Non-Secretor. (**a**) Korea; (**b**) Yanbian(China); (**c**) Pakistan and Vietnam; (**d**): All 4 countries.

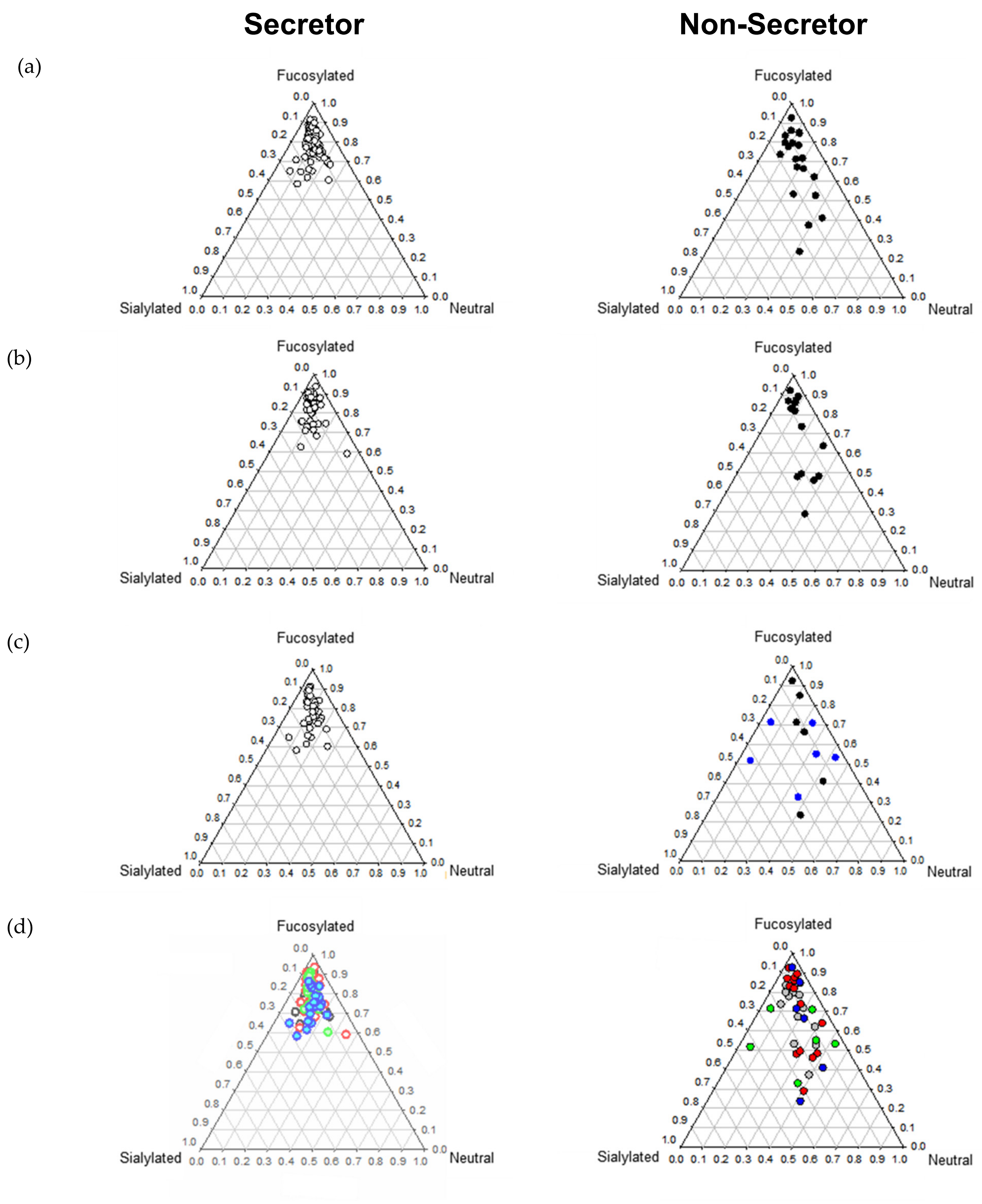

Supplementary Figure S2.

Percentile of HMOs in Secretor and Non-Secretor by glycan group; (**a**). Korea; (**b**) Yanbian(China)

(a)

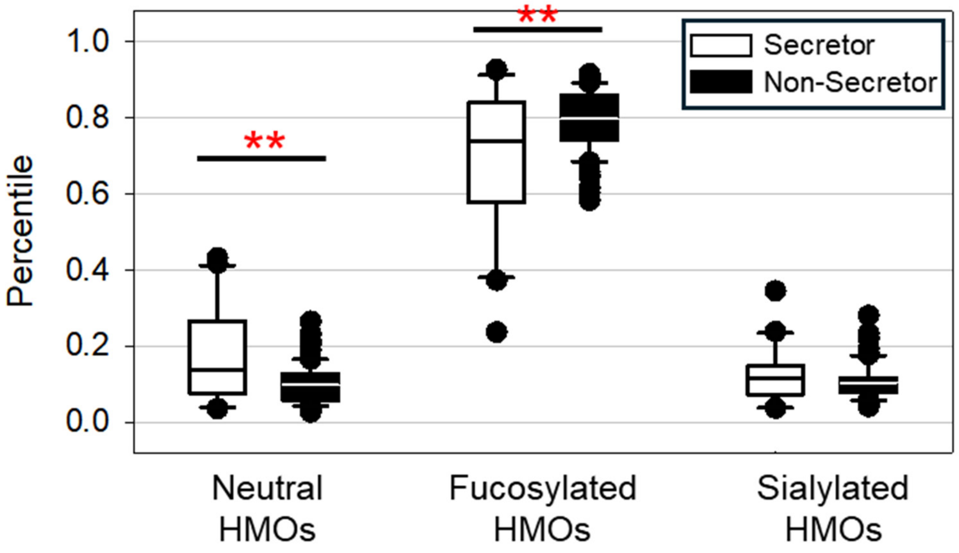

(b)

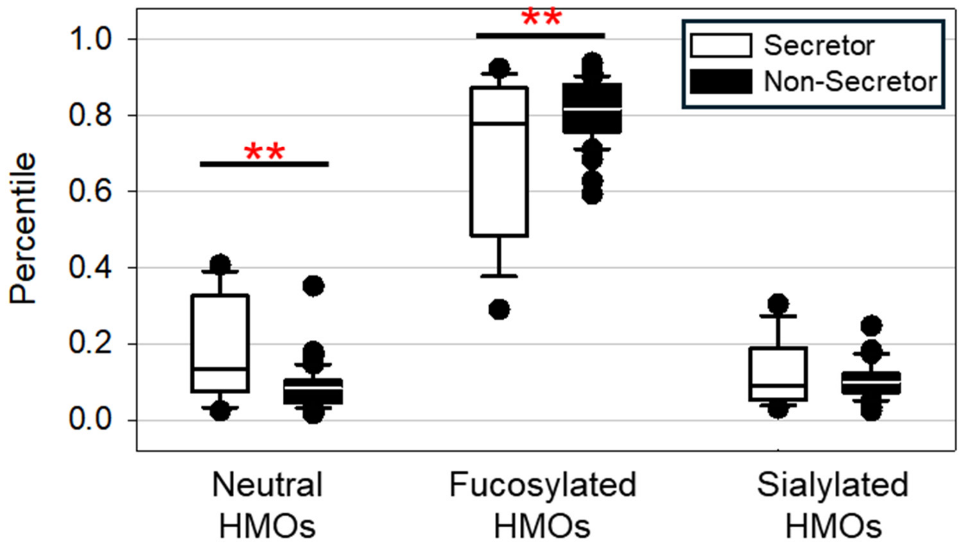

Supplementary Figure S3.

Box-and-Whisker plots of 15 Major HMOs from Secretor(Black box) and Non-Secretor(White box) in Asian human milk (mg/L) using sensitive QQQ quantitation. (a) Korea; (b) Yanbian(China); (c) Vietnam; (d) Pakistan.

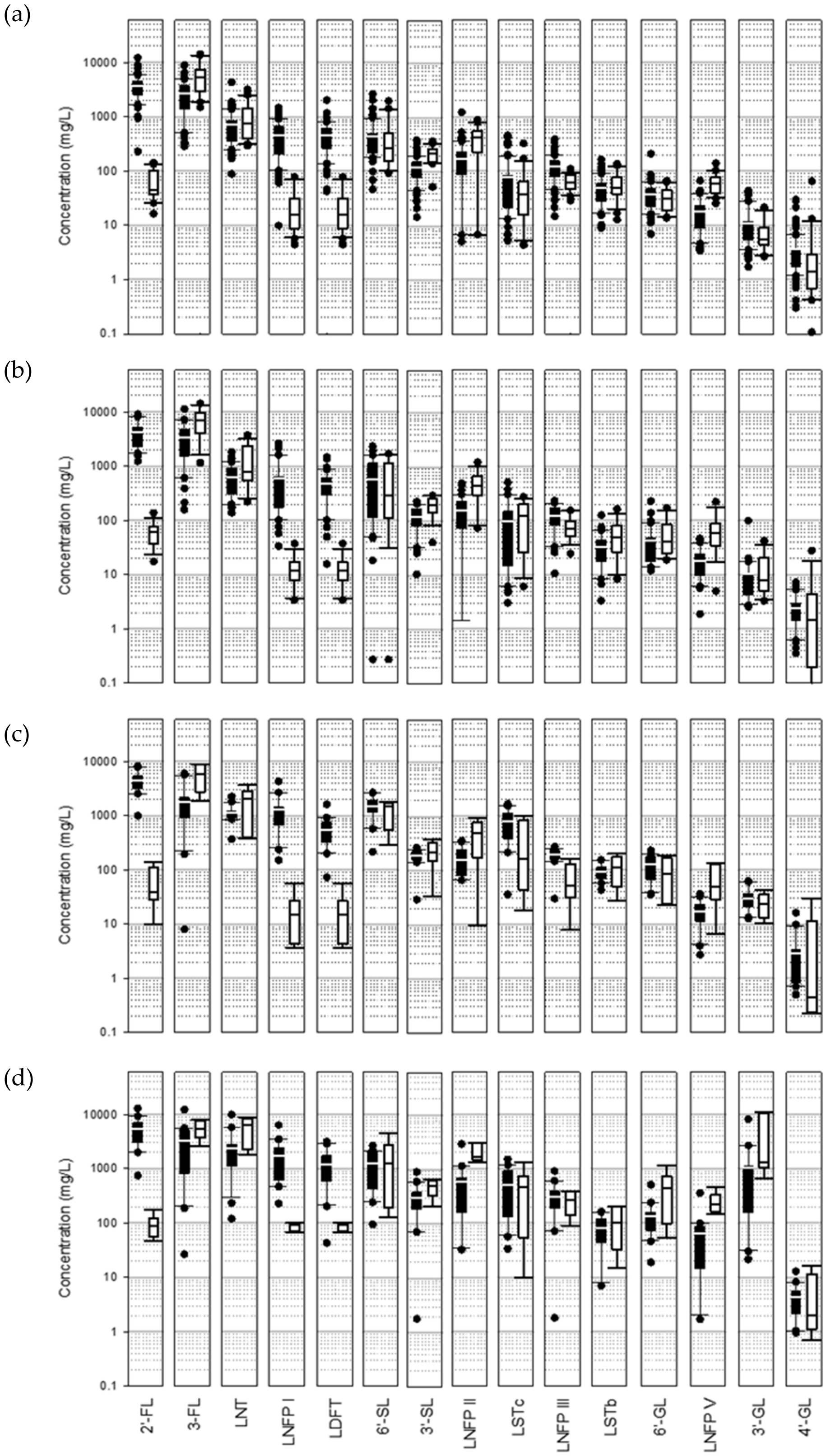

Supplementary Figure S4.

Quadrant scatter plots of specific HMO classes in human milk.
(**a**) Absolute value; (**b**) Z-value normalization.

(a)

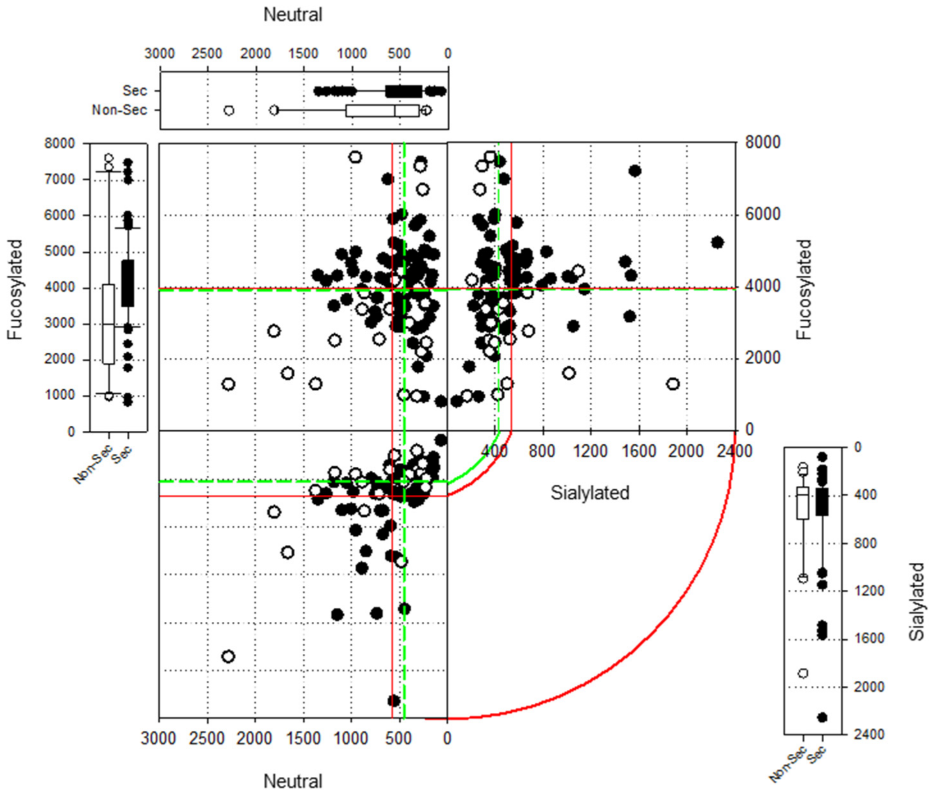

(b)

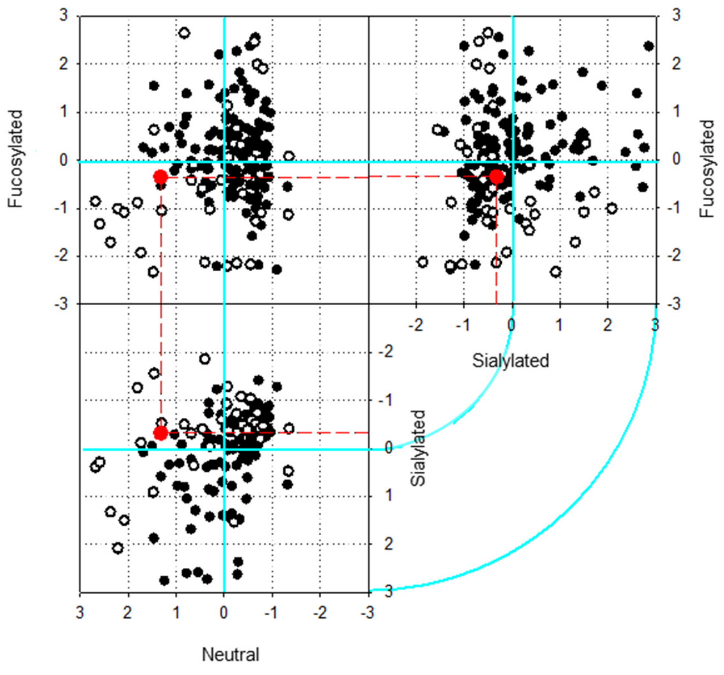

Supplementary Figure S5.

Conceptual representation of HMO biosynthetic relationships based on observed compositional patterns. (a) Neutral and sialylated HMOs; (b) Fucosylated HMOs; (c) Fucosylated and sialylated HMOs.

This schematic summarizes relationships inferred from quantitative and compositional data obtained in this study and is intended to provide a conceptual overview rather than a definitive biosynthetic pathway. Structures highlighted in bold font indicate those that were reduced or not detected in non-secretor phenotypes. Specifically, bold red text denotes structures supported by quantitative analytical data, whereas bold green text indicates structures inferred based on the presence of α-1,2-fucosylated linkages within the corresponding structures.

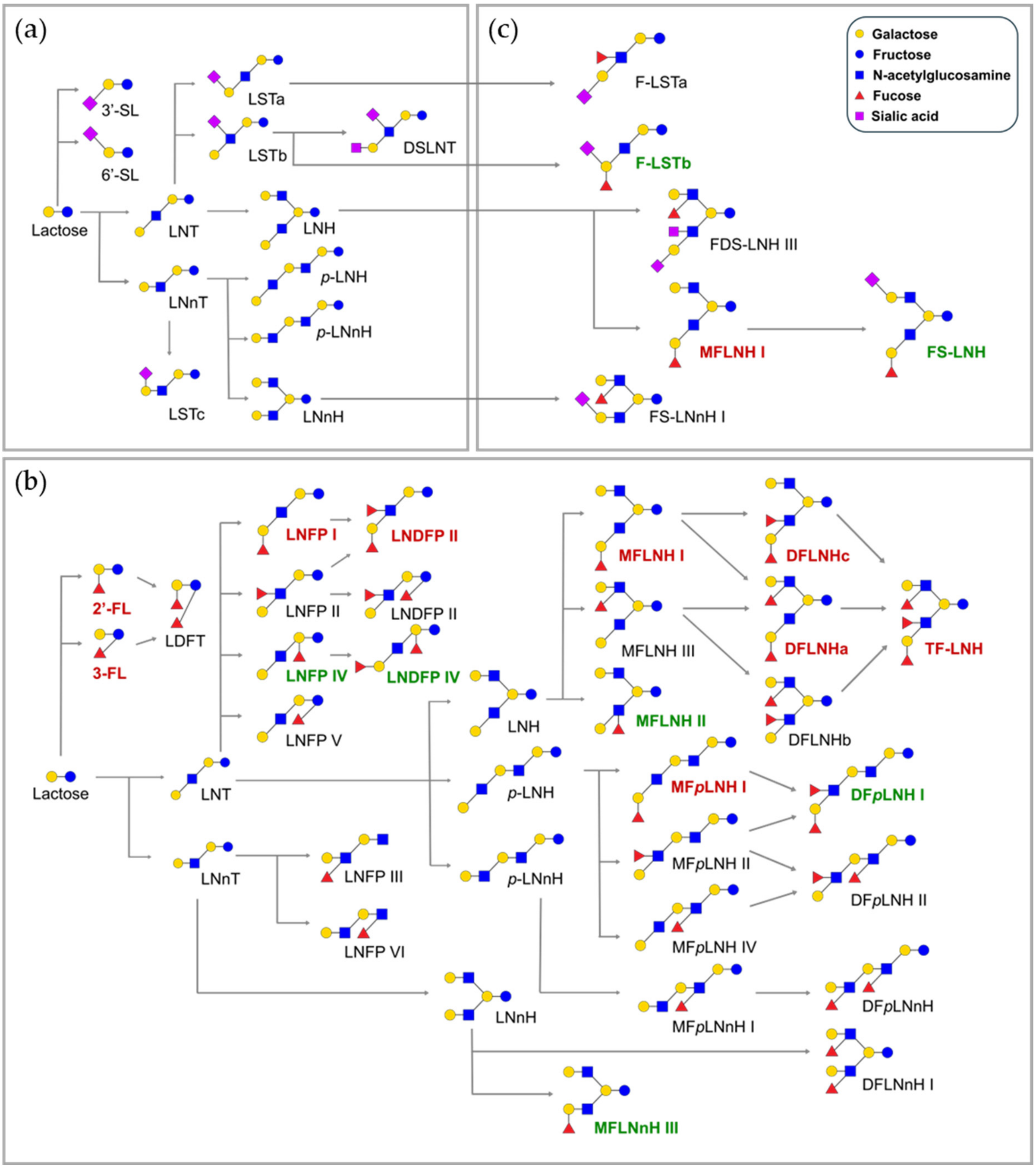

Supplementary Table S1.

Table S1. General characteristics of lactating mothers and their infants.

	KOREA	YANBIAN (CHINA)	VIETNAM	PAKISTAN
Mothers (*n*)	252	137	92	97
**Age (years)**	32.7 ± 3.3[c*]	30.5 ± 3.5[b]	28.9 ± 5.1[a]	28.4 ± 5.3[a]
**Body mass index (kg/m²)**	21.8 ± 3.4[a]	22.4 ± 3.7[ab]	23.5 ± 3.1[b]	24.7 ± 4.6[c]
**Smoking (%)**				
Non/Indirect	82/18	50/50	52.9/47.1	100/0
**Parturition (%)**				
Vaginal/Cesarean	65.9/34.1	41.3/58.7	52.9/47.1	45.0/55.0
Infants				
**Age (days)**	111.8 ± 68.6[b]	132.5 ± 104.0[b]	43.2 ± 64.1[a]	46.1 ± 46.1[a]
**Sex (%)**				
Male	53.2	55.3	54.3	62.0
Female	46.8	44.7	45.7	38.0
**Weight (kg)**	6.9 ± 4.0[c]	6.8 ± 2.2[b]	4.3 ± 1.7[b]	2.9 ± 0.8[a]
**Height (cm)**	62.3 ± 9.5[b]	63.0 ± 7.4[b]	55.4 ± 7.7[b]	52.3 ± 3.7[a]

* Value is expressed as mean ± standard deviation. The different letters in a row indicate the significant differences between four countries at *$p < 0.05$ by Scheffe's multiple range tests.

Supplementary Table S2.

Table S2. The MS/MS parameter and MRM transitions of analytes in positive mode.

Compound Name	RT (min)	Prec Ion (m/z)	Prod Ion (m/z)	Dwell (ms)	Frag (V)	CE (V)	Cell Acc (V)	Polarity
LSTb	19.9	1001.3	366.1	40	100	15	5	Positive
LSTc	18.5	1001.3	366.1	40	100	30	5	Positive
LNFP I	10.6	856.2	204.1	40	100	15	5	Positive
LNFP II	7.5	856.2	204.1	40	100	15	5	Positive
LNFP III	7.0	856.2	204.1	40	100	15	5	Positive
LNFP V	11.1	856.2	204.1	40	100	15	5	Positive
LNT	11.2	710.2	366.1	40	100	10	5	Positive
LDFT	11.2	637.2	183.1	40	100	10	5	Positive
3'-SL	20.0	636.3	292.1	40	110	10	5	Positive
6'-SL	12.1	636.3	292.1	40	110	20	5	Positive
3'-GL	7.6	507.2	183.1	40	100	5	5	Positive
4'-GL	4.8	507.2	183.1	40	100	5	5	Positive
6'-GL	7.1	507.2	183.1	40	100	10	5	Positive
3-FL	2.8	491.2	183.1	40	100	4	5	Positive
2'-FL	8.9	491.2	183.1	40	100	10	5	Positive

* Abbreviations: RT, retention time; Prec Ion, precursor ion; Prod Ion, product ion; Dwell, dwell time; Frag, fragmentor voltage; CE, collision energy; Cell Acc, cell accelerator voltage.

Supplementary Table S3.

Table S3. Pearson correlation coefficients between HMOs concentrations in Korean human milk (n = 102).

	4'-GL	6'-GL	3'-GL	LNT	2'-FL	3-FL	LDFT	LNFP I	LNFP II	LNFP III	LNFP V	3'-SL	6'-SL	LSTb	LSTc
**4'-GL**	1.00														
**6'-GL**	0.13	1.00													
**3'-GL**	0.28	0.72	1.00												
**LNT**	0.09	0.63	0.40	1.00											
**2'-FL**	-0.05	0.39	0.30	-0.04	1.00										
**3-FL**	0.26	-0.06	-0.09	-0.09	-0.50	1.00									
**LDFT**	0.02	0.10	0.19	-0.22	0.36	0.05	1.00								
**LNFP I**	-0.02	0.33	0.45	0.22	0.75	-0.54	0.08	1.00							
**LNFP II**	0.20	0.44	0.15	0.54	-0.33	0.49	-0.13	-0.28	1.00						
**LNFP III**	0.02	0.52	0.41	0.40	0.34	0.02	0.65	0.29	0.32	1.00					
**LNFP V**	0.17	0.10	-0.01	0.51	-0.62	0.43	-0.32	-0.44	0.69	-0.05	1.00				
**3'-SL**	0.03	0.51	0.33	0.48	0.06	0.00	-0.08	0.14	0.25	0.25	0.18	1.00			
**6'-SL**	0.00	0.47	0.54	0.38	0.11	-0.16	-0.03	0.25	0.05	0.14	0.11	0.37	1.00		
**LSTb**	0.28	0.51	0.51	0.73	-0.02	0.11	0.00	0.27	0.45	0.43	0.45	0.40	0.28	1.00	
**LSTc**	0.04	0.65	0.68	0.53	0.33	-0.26	0.04	0.44	0.14	0.37	0.00	0.31	0.66	0.42	1.00

Supplementary Table S4.

Table S4. Pearson correlation coefficients between HMOs concentrations in Yanbian(China) human milk (n = 54).

	4'-GL	6'-GL	3'-GL	LNT	2'-FL	3-FL	LDFT	LNFP I	LNFP II	LNFP III	LNFP V	3'-SL	6'-SL	LSTb	LSTc
**4'-GL**	1.00														
**6'-GL**	0.25	1.00													
**3'-GL**	0.33	0.80	1.00												
**LNT**	0.11	0.66	0.36	1.00											
**2'-FL**	-0.04	0.28	0.23	-0.19	1.00										
**3-FL**	0.38	-0.31	-0.13	-0.17	-0.54	1.00									
**LDFT**	-0.16	-0.17	-0.04	-0.34	0.32	-0.02	1.00								
**LNFP I**	0.03	0.47	0.31	0.06	0.82	-0.57	0.03	1.00							
**LNFP II**	0.00	0.40	0.25	0.73	-0.43	0.26	-0.09	-0.30	1.00						
**LNFP III**	0.04	0.32	0.26	0.19	0.37	-0.11	0.69	0.27	0.25	1.00					
**LNFP V**	0.04	0.44	0.27	0.83	-0.46	0.15	-0.27	-0.29	0.92	0.09	1.00				
**3'-SL**	0.07	0.03	0.12	0.08	0.09	0.09	0.21	-0.03	0.36	0.18	0.20	1.00			
**6'-SL**	0.10	0.78	0.59	0.60	0.49	-0.48	-0.12	0.64	0.23	0.32	0.28	0.07	1.00		
**LSTb**	0.57	0.30	0.31	0.49	-0.22	0.22	-0.05	-0.08	0.43	0.21	0.45	0.25	0.29	1.00	
**LSTc**	-0.16	-0.15	-0.12	-0.01	-0.10	0.03	0.22	-0.19	0.00	0.10	0.00	-0.11	0.02	-0.04	1.00

Supplementary Table S5.

Table S5. Pearson correlation coefficients between HMOs concentrations in Pakistani human milk (n = 26).

	4'-GL	6'-GL	3'-GL	LNT	2'-FL	3-FL	LDFT	LNFP I	LNFP II	LNFP III	LNFP V	3'-SL	6'-SL	LSTb	LSTc
**4'-GL**	1.00														
**6'-GL**	-0.18	1.00													
**3'-GL**	0.39	0.10	1.00												
**LNT**	0.09	0.73	0.24	1.00											
**2'-FL**	0.06	-0.15	-0.24	0.00	1.00										
**3-FL**	0.34	0.06	0.32	0.22	-0.30	1.00									
**LDFT**	0.03	-0.29	-0.17	-0.25	0.44	-0.02	1.00								
**LNFP I**	-0.05	0.00	-0.27	0.08	0.85	-0.39	0.09	1.00							
**LNFP II**	0.24	0.59	0.49	0.85	-0.32	0.50	-0.38	-0.28	1.00						
**LNFP III**	0.21	0.26	0.01	0.62	0.48	0.20	0.37	0.32	0.38	1.00					
**LNFP V**	0.24	0.56	0.55	0.87	-0.30	0.40	-0.34	-0.26	0.97	0.40	1.00				
**3'-SL**	-0.20	0.37	-0.07	0.24	0.17	0.19	0.06	0.26	0.11	0.20	0.11	1.00			
**6'-SL**	-0.06	0.27	0.14	0.34	0.03	0.07	-0.12	0.21	0.24	0.04	0.32	0.54	1.00		
**LSTb**	-0.08	0.38	0.18	0.62	0.02	0.25	-0.05	0.07	0.53	0.40	0.60	0.69	0.74	1.00	
**LSTc**	-0.36	0.27	-0.29	0.15	0.26	-0.27	-0.12	0.49	-0.11	0.02	-0.08	0.71	0.69	0.58	1.00

Supplementary Table S6.

Table S6. Pearson correlation coefficients between HMOs concentrations in Vietnamese human milk (n = 26).

	4'-GL	6'-GL	3'-GL	LNT	2'-FL	3-FL	LDFT	LNFP I	LNFP II	LNFP III	LNFP V	3'-SL	6'-SL	LSTb	LSTc
**4'-GL**	1.00														
**6'-GL**	0.20	1.00													
**3'-GL**	0.11	0.64	1.00												
**LNT**	0.59	0.33	0.15	1.00											
**2'-FL**	-0.24	0.43	0.27	-0.38	1.00										
**3-FL**	-0.06	-0.30	-0.17	0.16	-0.70	1.00									
**LDFT**	-0.18	0.22	0.26	-0.45	0.52	-0.50	1.00								
**LNFP I**	-0.15	0.43	0.21	-0.20	0.87	-0.59	0.16	1.00							
**LNFP II**	0.32	-0.15	-0.05	0.55	-0.66	0.47	-0.43	-0.53	1.00						
**LNFP III**	0.05	0.42	0.35	-0.10	0.51	-0.49	0.68	0.21	-0.19	1.00					
**LNFP V**	0.44	-0.13	-0.07	0.62	-0.59	0.46	-0.44	-0.47	0.94	-0.21	1.00				
**3'-SL**	-0.04	0.15	-0.04	-0.15	0.28	-0.29	0.41	0.22	-0.25	0.36	-0.25	1.00			
**6'-SL**	0.09	0.18	0.03	-0.22	0.44	-0.53	0.44	0.31	-0.36	0.37	-0.25	0.32	1.00		
**LSTb**	0.22	-0.27	-0.35	-0.21	-0.18	0.21	-0.11	-0.21	0.02	-0.06	0.18	-0.11	0.16	1.00	
**LSTc**	0.01	0.36	0.30	-0.10	0.50	-0.58	0.33	0.44	-0.29	0.46	-0.24	0.44	0.71	-0.15	1.00
